# Persistent DNA strand breaks induce a CAF-like phenotype in normal fibroblasts

**DOI:** 10.18632/oncotarget.24446

**Published:** 2018-02-07

**Authors:** Arnaud J. Legrand, Mattia Poletto, Daniela Pankova, Elena Clementi, John Moore, Francesc Castro-Giner, Anderson J. Ryan, Eric O’Neill, Enni Markkanen, Grigory L. Dianov

**Affiliations:** ^1^ CRUK & MRC Oxford Institute for Radiation Oncology, University of Oxford, Department of Oncology, Old Road Campus Research Building, OX37DQ Oxford, UK; ^2^ Institute of Pharmacology and Toxicology, Vetsuisse Faculty, University of Zürich, Zürich 8057, Switzerland; ^3^ Functional Genomics Center Zürich, University of Zürich, Zürich 8057, Switzerland; ^4^ Institute of Cytology and Genetics, Russian Academy of Sciences, Novosibirsk 630090, Russian Federation; ^5^ Novosibirsk State University, Novosibirsk 630090, Russian Federation

**Keywords:** tumour microenvironment, cancer-associated fibroblasts, base excision repair, tumour stroma, midostaurin

## Abstract

Cancer-associated fibroblasts (CAFs) are an emerging target for cancer therapy as they promote tumour growth and metastatic potential. However, CAF targeting is complicated by the lack of knowledge-based strategies aiming to selectively eliminate these cells. There is a growing body of evidence suggesting that a pro-inflammatory microenvironment (e.g. ROS and cytokines) promotes CAF formation during tumorigenesis, although the exact mechanisms involved remain unclear. In this study, we reveal that a prolonged pro-inflammatory stimulation causes a *de facto* deficiency in base excision repair, generating unrepaired DNA strand breaks and thereby triggering an ATF4-dependent reprogramming of normal fibroblasts into CAF-like cells. Based on the phenotype of *in vitro*-generated CAFs, we demonstrate that midostaurin, a clinically relevant compound, selectively eliminates CAF-like cells deficient in base excision repair and prevents their stimulatory role in cancer cell growth and migration.

## INTRODUCTION

Cancer cells heavily depend on a microenvironment that sustains their metabolism and proliferation, promotes their survival and ensures their ability to migrate and invade the surrounding tissue [1]. One main component of the tumour microenvironment is the stromal fibroblast, often referred to as cancer-associated fibroblast (CAF) [1]. CAFs are generally described as “activated fibroblasts”, hardly distinguishable from myofibroblasts [2] that have undergone trans-differentiation and show increased expression of specific markers (e.g. α-smooth muscle actin (α-SMA), fibroblast activation protein (FAP), secreted protein acidic and rich in cysteine (SPARC) or platelet-derived growth factor receptor β (PDGFRβ)) [3], accompanied by a pro-inflammatory secretory signature [4] and an enhanced extra-cellular matrix (ECM) remodelling capacity [2].

Understanding the molecular events driving fibroblast trans-differentiation is of great importance for developing new strategies for cancer treatment and for identifying novel anticancer drugs. It is well established that CAF genesis is mediated by inflammation through cytokines (e.g. transforming growth factor β (TGFβ)) and/or reactive oxygen species (ROS) [5, 6]. However, it remains unclear how TGFβ or ROS may condition fibroblasts and drive their change into CAFs on a molecular basis. Importantly, ROS can target DNA and induce a wide range of oxidative DNA lesions [7], which are usually eliminated by the base excision repair (BER) pathway [8]. BER is a robust DNA repair pathway that processes multiple endogenous DNA lesions including base damage and DNA single strand breaks (SSBs), as well as acute DNA damage caused by many mutagens. However, despite the high capacity of BER, persistent exposure to DNA damaging agents might overload BER capacity and result in accumulation of unrepaired DNA damage [9]. Indeed, we have recently demonstrated that accumulation of unrepaired SSBs in normal human fibroblasts leads to a cellular response whose proteomic profile is reminiscent of that observed in CAFs and cancer cells [10]. Therefore, we hypothesised that persistent DNA damage induced by ROS within the tumour microenvironment may lead to accumulation of unrepaired DNA strand breaks and reprogramming of normal fibroblasts into CAFs.

In this study, we demonstrate that a prolonged pro-inflammatory response can cause a deficiency in BER. This leads to an accumulation of unrepaired SSBs and induces trans-differentiation of normal human fibroblasts into CAF-like cells. Based on these findings we identified midostaurin, a clinically relevant compound, as a drug that selectively eliminates BER deficiency generated CAF-like cells and prevents their stimulatory role in cancer cell growth and migration.

## RESULTS

### Persistent exposure of fibroblasts to ROS or TGFβ promotes a decrease in BER capacity and accumulation of unrepaired DNA strand breaks, leading to trans-differentiation into CAF-like cells

**Figure 1:**
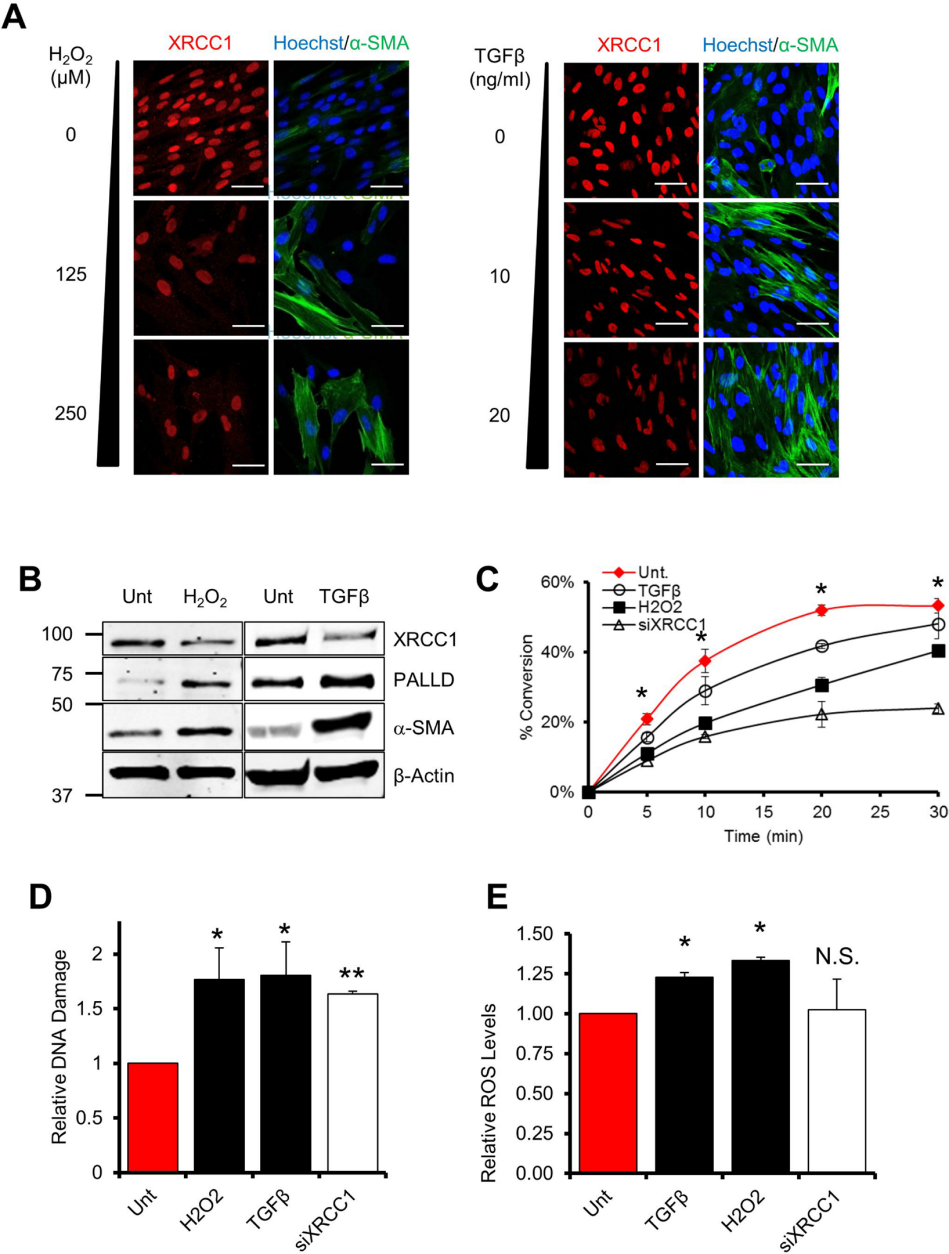
Persistent exposure of fibroblasts to ROS or TGFβ leads to a decrease of BER capacity (**A**–**B**) Effect of H2O2 or TGFβ on XRCC1, α-SMA and PALLD levels. (A) TIG-1 fibroblasts were treated for 72 h with H2O2 or TGFβ at the indicated concentrations, H2O2 was administered every 24 h. Cells were analysed by immunofluorescence using antibodies staining for α-SMA and XRCC1. Nuclei were stained with Hoechst. Scale bars: 50 μm. In (B) TIG-1 fibroblasts were treated for 72 h with either 125 μM H2O2 or 10 ng/ml TGFβ. Protein expression was analysed by Western blot. (**C**) Effect of H2O2 and TGFβ on BER capacity. Cells were treated as described in (B) and BER capacity was assessed by the *in vitro* repair assay using nuclear cell extracts generated from the indicated samples. The plot shows the percentage of substrate to product conversion over time using *in vitro* ligation assays as described in “Material and methods”. (**D**) Effect of H2O2 and TGFβ on DNA damage accumulation. TIG-1 fibroblasts were treated as in (B) or depleted for XRCC1 by means of siRNA. DNA damage accumulation was assessed 72h later using the alkaline comet assay. (**E**) Effect of TGFβ (10 ng/ml), H2O2 (20 μM), or XRCC1 depletion on the levels of intracellular ROS. TIG-1 fibroblasts were treated as indicated and analysed by FACS for intracellular ROS content. Data are reported as mean ± SD of three independent experiments ^*^p < 0.05; ^**^p < 0.01. See also Supplementary Figure 1.

Can unrepaired DNA strand breaks act as a trigger for trans-differentiation of normal fibroblasts? Tumours generally develop within a pro-inflammatory microenvironment, where a sustained release of pro-inflammatory and pro-fibrotic cytokines (e.g. TGFβ) by a variety of cell types accompanied by an increase in ROS production promote the generation of CAFs^5^. Accordingly, normal human TIG-1 fibroblasts treated for 72 hours with either H_2_O_2_ or TGFβ showed increased expression of markers such as α-SMA and PALLD, indicating that these experimental conditions induce an activated fibroblast signature (Figure 1A and 1B). Likewise, TGFβ treatment triggered an increase in the expression of the activated fibroblast markers, SPARC and FAP (Supplementary Figure 1A). Surprisingly, after treatment of the cells with H_2_O_2_ or TGFβ we also observed a concomitant loss of XRCC1–a BER protein essential for repair of SSBs arising due to endogenous mutagens [11] (Figure 1A and 1B). This decrease in XRCC1 was accompanied by an impaired BER capacity, as assessed by *in vitro* DNA repair assays, similar to what is observed after XRCC1 depletion by siRNA (Figure 1C). ROS are known to induce a variety of DNA lesions, including SSBs [7]; and over a prolonged exposure, could create a situation of persistent cellular stress and eventually exhaust BER capacity. With this in mind, we tested whether prolonged exposure of fibroblasts to either ROS or TGFβ could lead to an accumulation of unrepaired DNA damage. Indeed, persistent exposure of normal fibroblasts to H_2_O_2_ or TGFβ for 72 h led to accumulation of unrepaired DNA strand breaks, as measured by alkaline comet assays (Figure 1D) and by formation of 53BP1 foci (Supplementary Figure 1B). In addition, continuous exposure to TGFβ also resulted in an increase in intracellular ROS (Figure 1E), approximately equivalent to 18 mM H_2_O_2_ as calculated using a standard curve (Supplementary Figure 1C), thus explaining why fibroblasts treated with this pro-fibrotic cytokine showed accumulation of DNA damage. These results indicate that prolonged exposure to an oxidative microenvironment causes an exhaustion of BER that leads to accumulation of unrepaired DNA strand breaks in fibroblasts, concomitant with the induction of a CAF-like signature.

A large number of macromolecules can be modified by ROS. Our results suggested that, in the context of fibroblast trans-differentiation, ROS or DNA strand breaks could be a driving force for the emergence of CAFs. We thus wanted to address whether the exhaustion of BER could be a mechanism to amplify the stress response and trigger the trans-differentiation.

To answer these questions, we made use of a cellular model of BER deficiency generated by knockdown of expression of the XRCC1 protein that has been thoroughly characterised in our laboratory [10]. In this model, XRCC1 KD causes accumulation of SSBs in the absence of detectable DNA double strand breaks (DSBs) [12]. Interestingly, depletion of XRCC1 in normal human fibroblasts (hereafter XRCC1 KD cells) led to an accumulation of DNA damage comparable to a level observed when fibroblasts are treated with H_2_O_2_ or TGFβ (Figure 1D). Furthermore, in contrast to cells treated with H_2_O_2_ or TGFβ, XRCC1 KD fibroblasts did not show any increase in intracellular ROS (Figure 1E). This prompted us to exploit XRCC1 KD cells to investigate whether unrepaired SSBs alone, independently of DSBs or oxidative stress, can trigger trans-differentiation into CAF-like cells.

### XRCC1 depletion leads to ATF4-dependent reprogramming of normal fibroblasts and the emergence of a gene expression signature characteristic of CAFs

We have previously performed proteome-wide analysis on normal human fibroblasts depleted of XRCC1 [10]. This analysis suggested that the proteomic profile of XRCC1 KD cells was reminiscent of that usually observed in CAFs and tumour cells [10], indicating that fibroblasts experience major phenotypical rearrangements in response to persistent DNA damage.

**Figure 2:**
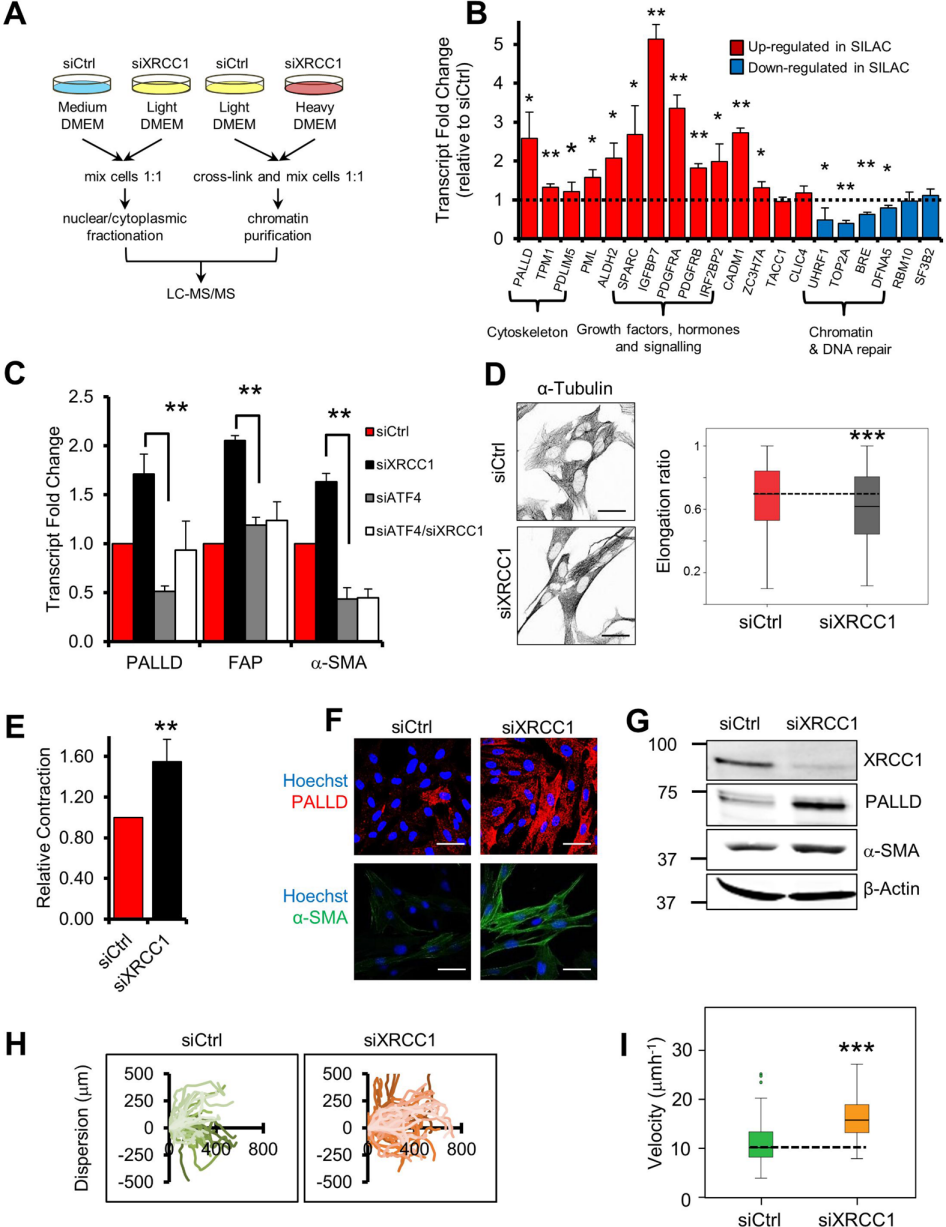
XRCC1 depletion leads to reprogramming of normal fibroblasts into CAF-like cells (**A**) Schematic representation of the SILAC-based proteomics analysis. (**B**) Validation of protein expression changes at the transcriptional level. TIG-1 fibroblasts were transfected with either a non-targeting control siRNA (siCtrl), or an XRCC1-targeting siRNA for 72 h and expression of the indicated genes was analysed by qPCR. The dashed line represents the normalised expression level in siCtrl-treated cells. (**C**) Rescue of the expression of PALLD, FAP and α-SMA upon simultaneous depletion of XRCC1 and ATF4. Samples were analysed by qPCR 72 h after siRNA transfection. (**D**) XRCC1 KD fibroblasts show increased cytoskeleton density and an elongated shape. Cells were stained for α-tubulin (left panel). Nuclei were stained with Hoechst. Scale bars: 50 μm. Cell elongation ratio (right panel) was assessed by IN Cell high-throughput imagery, as described in Materials and methods. Statistical significance was evaluated by a non-parametric Mann-Whitney *U* Test. (**E**) XRCC1 depletion leads to contraction of the cytoskeleton.TIG-1 fibroblasts were treated as in (B) and analysed for contractility. Relative contraction was assessed using a collagen gel-based assay, as described in Materials and methods. (**F**–**G**) XRCC1 depletion leads to increased expression of PALLD and α-SMA. TIG-1 fibroblasts were treated as in (B) and analysed by immunofluorescence (F) or western blotting (G). Cells were stained for PALLD and α-SMA. Nuclei were stained with Hoechst. Scale bars: 50 μm. (**H**–**I**) Effect of XRCC1 depletion on fibroblast migration. Cells were incubated with the indicated siRNA for 48 h before the wound was generated and monitored through live microscopy for 20 h. Individual cells were manually tracked and their dispersion (H) or velocity (I) was calculated. Results are presented as mean ± SD of at least three independent experiments ^*^p < 0.05; ^**^p < 0.01; ^***^p < 0.001.

To increase the resolution of our previous analysis we exploited the stable isotope labelling by amino acids in cell culture (SILAC) technology and specifically analysed the chromatin-bound proteome using the “chromatin enrichment for proteomics” (ChEP) methodology [13] (Figure 2A). Functional clustering of the merged data provided a landscape of the proteomic changes occurring in normal fibroblasts upon SSB accumulation induced by BER depletion. Strikingly, we observed major upregulation of proteins involved in cellular metabolism, signalling and ECM remodelling (Supplementary Table 1). These changes strongly resembled those typically found in CAFs [1, 5], with upregulation of palladin (PALLD), SPARC, caldesmon (CALD1), PDGF receptors (both α and β) and matrix metalloproteinase-2 (MMP2) (Supplementary Table 2).

To determine whether the observed proteomic profile was the consequence of a broad transcriptional reprogramming rather than differential protein stability, we analysed mRNA levels of a subset of hits identified in the SILAC analysis. For the majority of the selected genes deregulation was validated at the mRNA level, confirming that accumulation of unrepaired strand breaks, caused by XRCC1 depletion, induced a vast transcriptional reprogramming in fibroblasts (Figure 2B). In addition to the genes found in the SILAC analysis, we detected increased expression of FAP and α-SMA, as well as decreased levels of calveolin-1 (CAV1) (Supplementary Figure 2A and Supplementary Table 2), well-known CAF markers [5, 14]. Importantly, a similar gene expression profile was triggered by depletion of XRCC1 using different siRNA sequences (Supplementary Figure 2B) and in other fibroblast cell lines of foetal (Supplementary Figure 2C) and adult (Supplementary Figure 2D) origin, thus excluding both cell-line specific mechanisms and off-target effects of the XRCC1 siRNA.

We have previously demonstrated activation of the transcription factor ATF4 upon XRCC1 depletion [10]. Here, we confirmed increased ATF4 expression in XRCC1 KD cells (Supplementary Figure 2E). Furthermore, we found that simultaneous depletion of XRCC1 and ATF4 completely prevented the transcriptional upregulation of CAF markers such as FAP and α-SMA and partially the upregulation of PALLD, which were induced in XRCC1 KD fibroblasts (Figure 2C). ATF4 depletion itself caused a decrease in PALLD and α-SMA expression (Figure 2C), suggesting that it also controls the basal expression of these genes.

Altogether, these results suggest that accumulation of unrepaired DNA strand breaks in BER-deficient fibroblasts is sufficient to drive a transcriptional reprogramming resembling that usually observed in CAFs. This reprogramming is dependent on the activation of the stress-responsive transcription factor ATF4.

### XRCC1 KD fibroblasts display functional characteristics similar to CAFs

As XRCC1 KD cells showed transcriptional markers typical of CAFs, we sought to determine whether unrepaired SSBs could actually lead to a CAF-like phenotype. Phenotypical characterisation of XRCC1 KD fibroblasts by α-tubulin staining revealed a modified cytoskeleton with thick aligned fibres and an elongated cell shape, a distinctive characteristic of activated fibroblasts (Figures 2D and Supplementary Figure 2F) [15]. Accordingly, XRCC1 KD cells showed increased contractility, as measured by *in vitro* collagen contraction assays (Figure 2E), and this phenotype correlated with increases in PALLD and α-SMA protein levels (Figures 2F, 2G, Supplementary Figure 2G, and 2H), both of which promote cytoskeleton contraction and migration in CAFs [3, 16]. In line with these observations, XRCC1 KD cells showed increased migration, displaying greater dispersion (Figure 2H) and augmented velocity (Figure 2I), as assessed by single-cell tracking. Interestingly, these phenotypical changes were not transitory, as upon XRCC1 depletion PALLD failed to return to basal levels and cytoskeleton contraction persisted, even when XRCC1 expression was partially restored (Supplementary Figure 3A, 3B), suggesting terminal differentiation.

**Figure 3:**
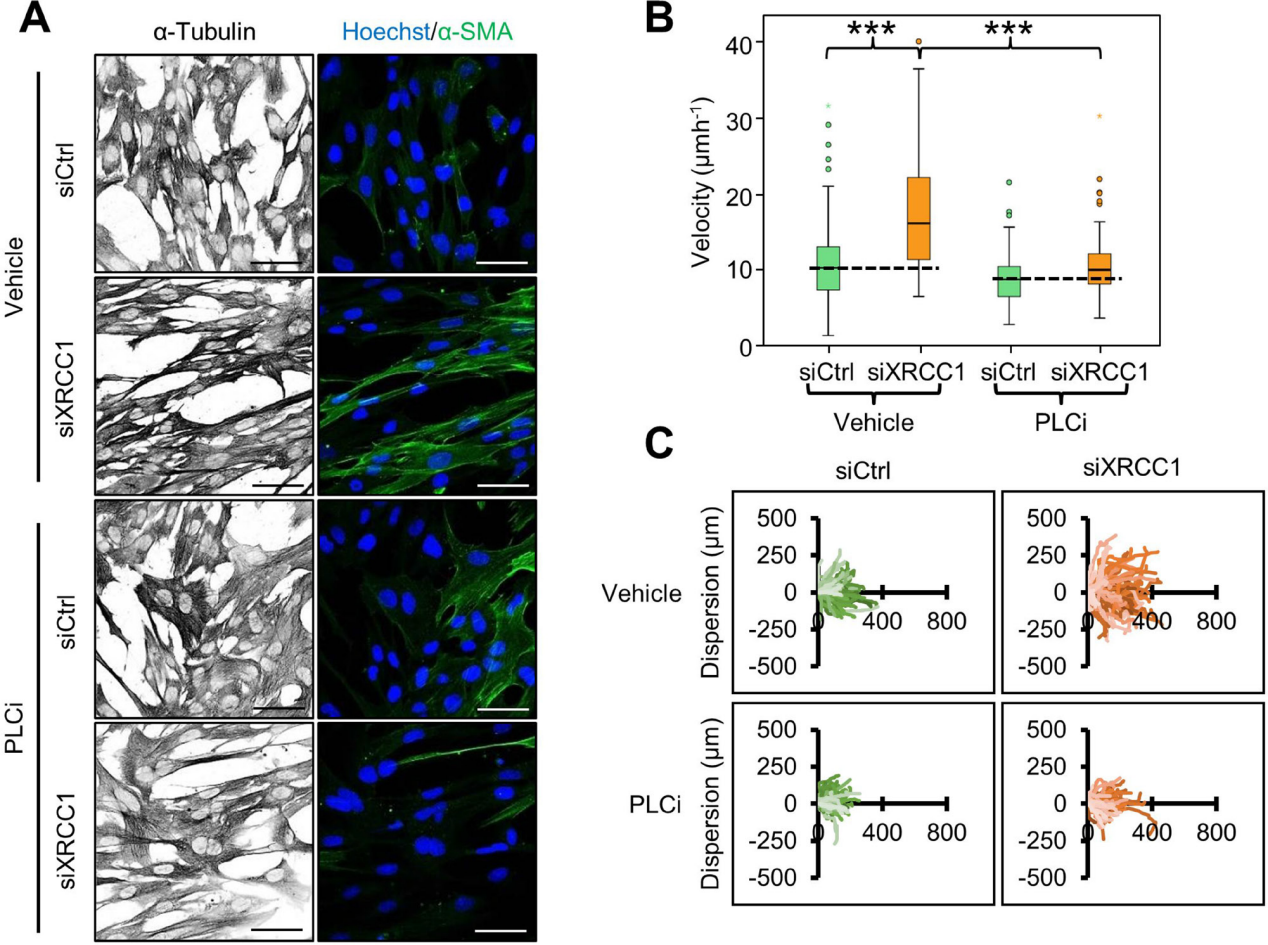
Calcium release is required for cytoskeleton contraction and migration of XRCC1 KD fibroblasts (**A**) Effect of PLC inhibition on cytoskeleton contraction. TIG-1 fibroblasts were treated with the indicated siRNA for 72 h. The PLC inhibitor edelfosine (PLCi, 10 μm) was added during the final 48 h of the experiment. Cells were subsequently stained for α-tubulin and α-SMA. Nuclei were stained with Hoechst. Scale bars: 50 μm. (**B**–**C**) Effect of simultaneous XRCC1 depletion and PLC inhibition on fibroblast migration. TIG-1 fibroblasts were treated with the indicated siRNA and incubated with PLCi 24 h before the wound was generated and the acquisition was carried out under live cell microscopy for 20h. Individual cells were manually tracked to analyse their velocity (B) and dispersion (C). ^***^p < 0.001.

Intracellular Ca^2+^ is essential for contraction of activated fibroblasts [17] and for the maintenance of their phenotype [18]. Accordingly, we found that suppression of Ca^2+^ release from the endoplasmic reticulum through inhibition of phospholipase C (PLC) activity led to relaxation of the cytoskeleton and decreased α-SMA staining in XRCC1 KD cells (Figure 3A), as well as prevention of the migratory phenotype (Figure 3B and 3C). These observations further support the activated fibroblast phenotype of XRCC1 KD cells.

**Figure 4:**
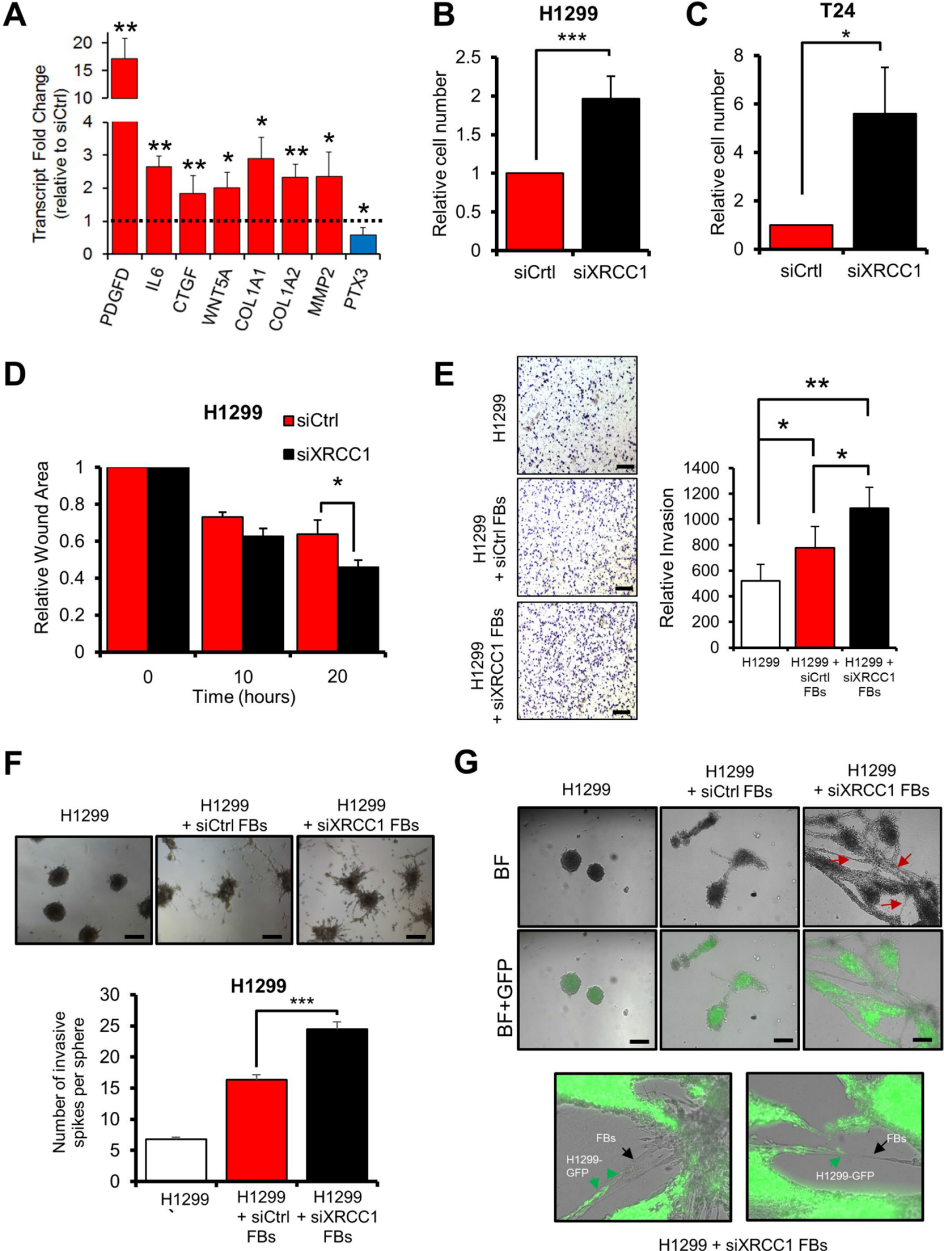
XRCC1 KD fibroblasts promote growth and migration of cancer cells (**A**) Effect of XRCC1 depletion on the expression of secreted proteins. TIG-1 fibroblasts were depleted of XRCC1 and expression of the indicated genes was analysed by qPCR. The dashed line represents the normalised expression level in cells treated with control siRNA. (**B**–**C**) Stimulation of proliferation of H1299 (B) or T24 (C) cells by medium conditioned by XRCC1 KD fibroblasts. TIG-1 fibroblasts were treated with the indicated siRNAs for 72 h, conditioned medium was then collected and used to feed cancer cells for five days. (**D**) Stimulation of migration of H1299 cells by medium conditioned by XRCC1 KD TIG-1 fibroblasts. Conditioned medium was generated as in (B), H1299 were then fed with the conditioned medium for 24 h before assessing their migration using a wound healing assay. Faster wound closure indicates higher migration capacity. (**E**) Increased invasion of H1299 cells in co-culture with XRCC1 KD fibroblasts. TIG-1 fibroblasts were treated with the indicated siRNAs for 48 h and then cultured together with H1299 cells in Boyden chambers in the presence of conditioned medium. Images were acquired 24h after cell seeding and invading cells were counted. Scale bars: 200 µm. (**F**) Stimulation of invasion of H1299 cells by XRCC1 KD fibroblasts. Cell spheroids were generated by culturing H1299 cells alone or by co-culturing H1299 cells together with TIG-1 fibroblasts (FBs) which were pretreated with the indicated siRNA. Two days after plating onto Matrigel, the spheroids were assessed for their tumorigenic potential by counting the number of invasive spikes (right). (**G**) Migration via network structure formation on three-dimensional Matrigel. H1299-GFP cancer spheroids were plated on a Matrigel matrix. After spheroid seeding, a single cell suspension of either control (siCtrl) or XRCC1 KD fibroblasts (FBs) was added on top of the spheroid culture and imaged 24 h later. siXRCC1-treated fibroblasts created a network characterised by H1299-GFP cancer spheroid invasion (red arrows). Enlargements of these networks are shown on the right panel: XRCC1 KD fibroblasts (black arrows) appear to open the way for H1299-GFP cells (green arrows). Scale bars: 100 µm. BF: Bright field. Representative fields are reported. Results are presented as mean ± SD of at least three independent experiments ^*^p < 0.05; ^***^p < 0.001.

Additionally, CAFs affect their microenvironment through secretion of molecules such as growth factors and cytokines [1, 5]. XRCC1 KD cells showed high expression of the growth factors PDGF-D and connective tissue growth factor (CTGF) as well as the cytokine interleukin-6 (IL-6) and attenuated expression of the tumour suppressor pentraxin-related protein PTX3, an important modulator of inflammation and immune response [19] (Figure 4A), all of which are changes characteristic of activated fibroblasts. Changes in several ECM components known to promote growth of tumour cells [1, 15], such as the collagens COL1A1, COL1A2, and the MMP2 metalloprotease (Figure 4A) were also evident in XRCC1 KD cells, again reflecting an activated fibroblast phenotype. In conclusion, trans-differentiation induced by BER deficiency in fibroblasts results in a CAF-like phenotype characterised by cytoskeleton contraction, increased migratory ability, and augmented expression of secreted molecules that can promote cell growth and migration.

### XRCC1 KD fibroblasts promote growth and invasion of cancer cells

The phenotype observed upon XRCC1 depletion suggests that, similarly to CAFs, XRCC1 KD cells might have tumour promoting properties. Thus, we assessed the ability of XRCC1 KD fibroblasts to support growth and motility of cancer cells. Notably, conditioned medium from two different lung-derived fibroblast lines with XRCC1 KD, significantly stimulated the growth of non-small cell lung carcinoma-derived H1299 cells (Figure 4B and Supplementary Figure 4A) and lung carcinoma-derived A549 (Supplementary Figure 4B) cell lines. Similar effects could be seen with the bladder carcinoma-derived T24 cell line (Figure 4C and Supplementary Figure 4C), suggesting that the phenomenon is not tissue-specific. Additionally, exposure of H1299 cells to medium conditioned by XRCC1 KD fibroblasts significantly enhanced their migratory properties (Figure 4D). Co-culturing XRCC1 KD fibroblasts and H1299 cells in Boyden chamber assays stimulated the invasive capacity of the cancer cells (Figure 4E). Similarly, when co-cultured as spheroids, XRCC1 KD fibroblasts significantly increased the invasive capability of H1299 cells (Figure 4F). Finally, migration assays on 3D matrix showed that XRCC1 KD fibroblasts were capable of forming network structures within the Matrigel matrix, supporting invasion of GFP-expressing H1299 cells out of the spheroids (Figure 4G).

Together, these data led us to conclude that unrepaired SSBs accumulating in BER-depleted cells can trigger the conversion of fibroblasts towards a CAF-like phenotype that supports growth, migration, invasion and tumorigenic capabilities of cancer cells.

### Analysis of clinical stroma samples support the link between BER dysfunction and CAF phenotype

The phenotypical changes induced in normal fibroblasts by XRCC1 depletion support the idea that BER impairment could accelerate accumulation of DNA strand breaks in a pro-inflammatory tumour microenvironment. Thus, by acting as a trigger for trans-differentiation, BER impairment could possibly constitute an actual risk factor for the emergence of CAFs.

**Figure 5:**
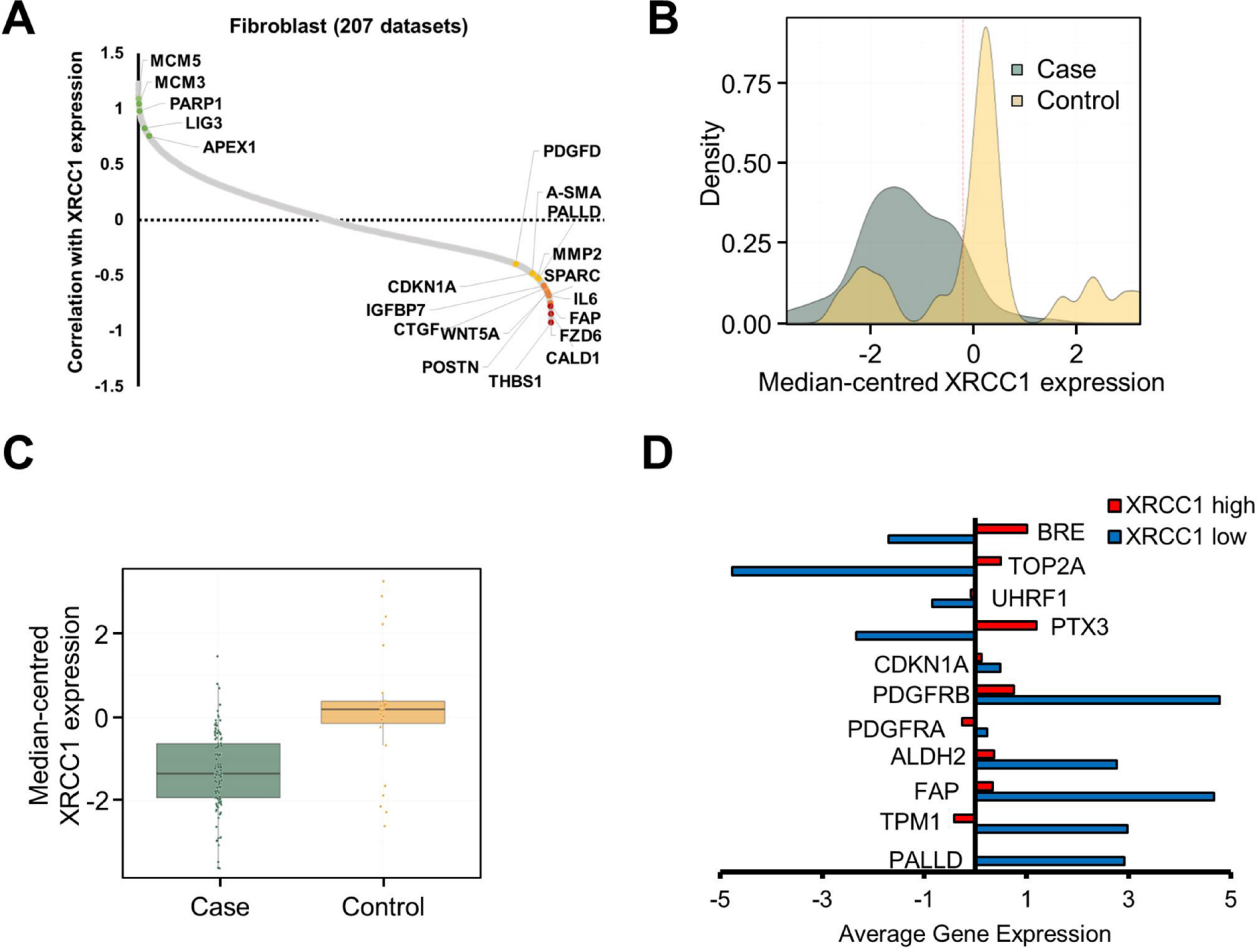
Expression of BER genes in clinical stroma samples negatively correlates with CAF markers (**A**) Plot showing the correlation between XRCC1 expression levels and the expression level of a number of human genes in 207 fibroblast datasets. BER and replication-associated genes (green dots) positively correlate with XRCC1 expression, whereas CAF markers (yellow and red dots) negatively correlate with XRCC1. Correlation is expressed as Z-score. A complete list of genes correlating with XRCC1 expression can be found in Supplementary Table 3. (**B**) Density plot depicting the distribution of tumour against control cases and the variation in XRCC1 expression within the samples analysed. The dashed line shows the arbitrary threshold selected to separate samples on the basis of XRCC1 expression level. Expression values correspond to the log2 median-centred values by sample. (**C**) Boxplot showing the median-centred expression of XRCC1 in tumour cases against controls using the threshold selected in (B). (**D**) Histogram showing the average expression of the indicated genes in low-XRCC1 vs. high-XRCC1 expressing samples.

In order to understand whether this could be observed *in vivo*, we carried out a series of bioinformatics analyses on gene co-expression studies performed on human tissue samples. A negative correlation between CAF markers and XRCC1 expression was observed when analysing datasets that uniquely consist of stromal samples (Supplementary Figure 5A and Supplementary Table 3). Strikingly, restricting the analysis to fibroblast-only containing datasets yielded a marked negative correlation between XRCC1 expression and CAF markers (Figure 5A and Supplementary Table 3). A Gene Ontology enrichment analysis of this fibroblast-only dataset revealed that genes which positively correlated with XRCC1 expression were mainly involved in splicing/transcription, as well as DNA repair and cell-cycle related processes (Table 1 and Supplementary Table 4). Conversely, genes negatively correlated with XRCC1 expression were clustered into tissue remodelling and wound healing processes (Table 1 and Supplementary Table 4). These findings are entirely consistent with the negative correlation between DNA repair and CAF markers shown in Figure 5A and indicate that a DNA repair deficiency *in vivo* could indeed be associated with appearance of a CAF-like phenotype.

**Table 1:** Gene Ontology (G.O.) analysis of proteins that positively or negatively correlate with XRCC1 expression in fibroblasts

Description	FDR *q* value	Enrichment
*Cell cycle process*	2.65E-87	5.75
*Mitotic cell cycle process*	1.12E-81	6.95
*Mitotic cell cycle*	2.29E-71	8.73
*Cell cycle*	8.88E-70	6.84
*DNA metabolic process*	1.94E-64	5.94
*Extracellular matrix organization*	5.79E-07	3.56
*Extracellular structure organization*	3.14E-07	3.55
*Vesicle-mediated transport*	9.29E-04	2.05
*Collagen fibril organization*	2.29E-03	8.60
*Regulation of cell migration*	2.09E-03	2.26

The top five G.O. terms that positively (lines 1–5) or negatively (lines 6–10) correlate with XRCC1 expression are shown. The full list of G.O. terms is provided in Supplementary Table 4.

To investigate whether the same features could be observed in samples from tumour stroma gathered from clinical studies, two independent breast cancer datasets with a wide distribution of XRCC1 expression levels were analysed [20, 21] (Figure 5B). Comparison between tumour and control samples showed a substantial downregulation of XRCC1 in the tumour stroma (Figure 5C). Interestingly, in XRCC1-low samples an increased expression of CAF markers and a concomitantly decreased expression of chromatin remodelling and DNA repair genes (e.g. UHRF1, TOP2A and BRE) was found (Figure 5D and Supplementary Figure 5B), validating the results obtained by our proteomics analysis. Importantly, when samples were separated into case and controls before the analysis, the expression pattern characterising XRCC1-low samples was preserved in tumour samples (Supplementary Figure 5C), suggesting that lower XRCC1 expression could potentially be used as prognostic indicator for tumours that are enriched in CAFs.

Altogether, these data show that XRCC1 downregulation is associated with the emergence of CAF markers in human tumour stroma samples and fully confirm the observations we made *in vitro*.

### Midostaurin sensitises BER deficient CAF-like cells to apoptosis

**Figure 6:**
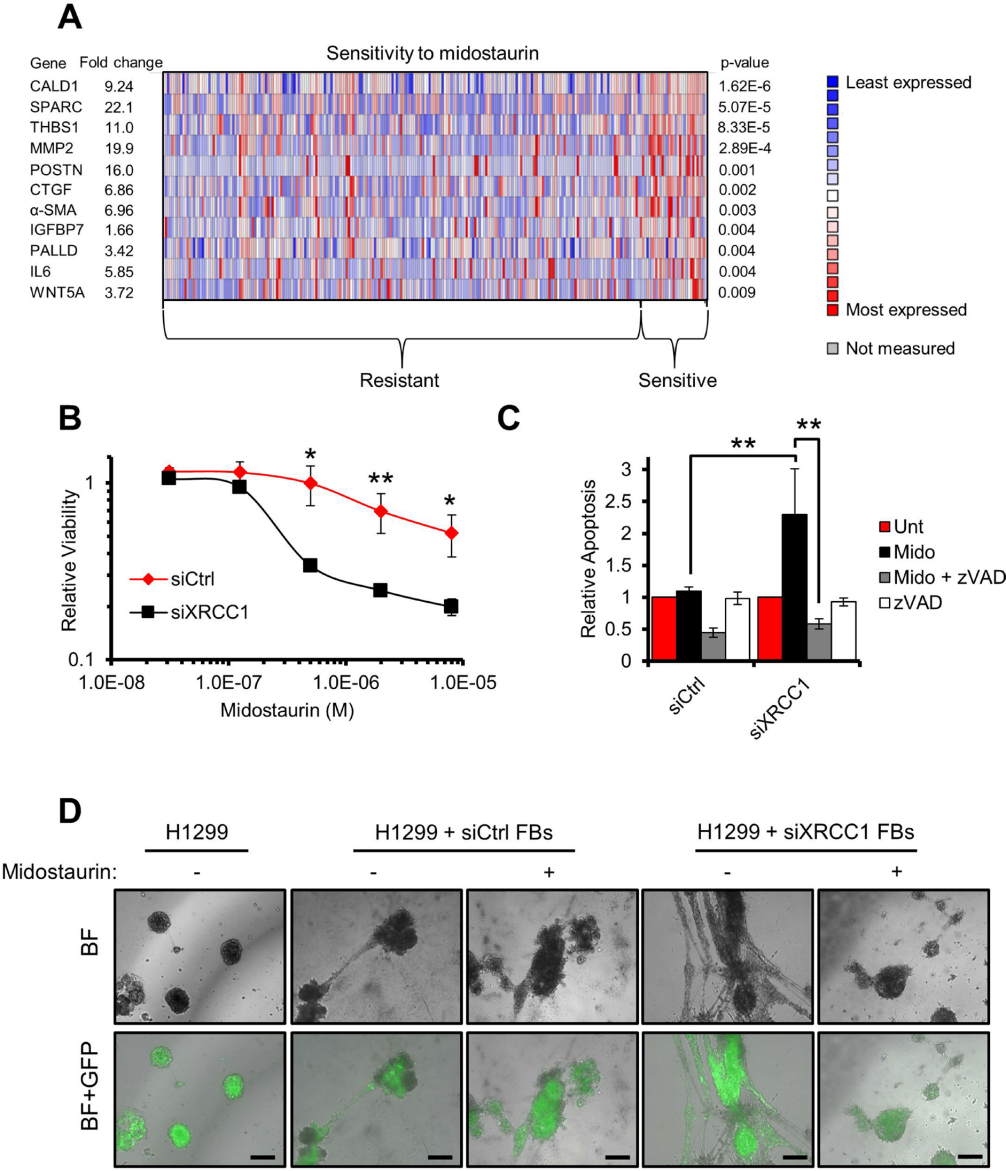
Midostaurin eliminates XRCC1 KD fibroblasts and negatively affects their stimulatory ability towards cancer cells (**A**) Heat-map showing the expression of the genes selected from a “BER/CAF” signature in both midostaurin-resistant and -sensitive cell lines. Each column represents an individual cell line. The mean differential gene expression between midostaurin-resistant and -sensitive cell lines is reported for each individual gene of interest as a fold change (left-hand side). (**B**) Sensitivity of XRCC1 KD fibroblasts to midostaurin. TIG-1 fibroblasts were treated with the indicated siRNA for 48 h before exposure to increasing concentrations of midostaurin for 72 h. Cell viability was assessed using resazurin. (**C**) Increased apoptosis in XRCC1 KD fibroblasts after exposure to midostaurin. TIG-1 fibroblasts were treated as in (B). Caspase activity was assessed upon incubation with midostaurin (8 μM, 48 h). Specificity of the assay was confirmed by co-incubation of the drugs with the pan-caspase inhibitor zVAD. (**D**) Migration via network structure formation on three-dimensional Matrigel. H1299-GFP cancer spheroids were seeded on Matrigel. Either control (siCtrl) or XRCC1 KD fibroblasts (FBs) were then added on top of the spheroid culture and imaged 24 h later. Midostaurin (1 μM) was used to pre-treat the fibroblasts and then administered to the spheroids for a further 24 h. Scale bars: 100 µm. BF: Bright field. Representative fields are reported.

Finally, to investigate whether the information gathered by studying the XRCC1 KD model could be exploited to identify drugs targeting CAFs, we defined a “BER/CAF” signature encompassing the CAF markers consistently upregulated in response to BER-deficiency (Figures 5A and 6A). Using this signature and the Oncomine™ database, we interrogated a drug sensitivity dataset [22] and identified midostaurin as a potential candidate targeting low-XRCC1 cells (Figure 6A). Midostaurin is a staurosporine derivative currently under investigation in several clinical trials (www.clinicaltrials.gov). Staurosporines are potent multi-kinase inhibitors and can interfere with multiple functions essential to CAFs such as Ca^2+^ signalling [23] (Figure 3). Consistent with bioinformatics predictions, XRCC1 KD fibroblasts showed hypersensitivity to midostaurin (Figure 6B and Supplementary Figure 6A). They were also sensitive to staurosporine (Supplementary Figure 6B) and to its analogue UCN-01 (Supplementary Figure 6D). Consistent with cell viability data, caspase activity assays confirmed that XRCC1 KD fibroblasts were greatly sensitised to apoptosis induced by midostaurin and other staurosporines; as expected, caspase activation was efficiently rescued by the broad caspase inhibitor ZVAD (Figure 6C, Supplementary Figure 6C and 6E). Lastly, midostaurin completely prevented the stimulatory ability of XRCC1 KD fibroblasts towards cancer cells, when delivered to XRCC1 KD/H1299 co-cultured spheroids (Figure 6D). Interestingly, H1299 cells were not particularly sensitive to midostaurin, suggesting that the drug indeed targeted CAFs (Supplementary Figure 6F).

This evidence indicates that XRCC1 KD CAF-like cells are hypersensitive to midostaurin, which is also effective in preventing their tumour promoting capability.

## DISCUSSION

Growth and invasion of cancer cells strongly depends on contributions from the surrounding stroma, which modifies and shapes the tumour microenvironment through the action of CAFs [14]. For this reason, finding drugs that specifically eliminate CAFs is a major focus for the development of anti-cancer therapies [24, 25]. We previously reported that proteomic changes in BER-deficient fibroblasts are highly similar to those observed in CAFs and tumours [10]. Therefore, we hypothesised that the accumulation of unrepaired DNA strand breaks, a consequence of BER impairment, may constitute a mechanism underlying fibroblast reprogramming.

Several explanations have been put forward to elucidate the origin of CAFs and how they maintain their phenotype, implicating genetic mutations, epigenetic modifications and persistent environmental effects [2, 15]. While the consequences of mutations and epigenetic modifications are still under investigation, the effect of persistent exposures to TGFβ or ROS have been well-documented to cause the trans-differentiation of resident fibroblasts into CAFs [5, 6]. In this study, we now demonstrate that the role of DNA damage in this phenomenon has been largely overlooked.

Inflammation is a crucial feature in cancer [5, 26]. This complex phenomenon is characterised by the secretion of cytokines and a significant production of ROS [27]. We show here that fibroblasts persistently exposed to ROS or TGFβ do indeed accumulate unrepaired DNA lesions. Although the ability of ROS to generate DNA lesions is widely acknowledged [8], cytokines such as TGFβ are also capable of inducing oxidative stress and DNA damage [28–30]. This study confirms these observations in fibroblasts and takes the concept a step further, showing that long term exposure to ROS and TGFβ can trigger fibroblast trans-differentiation by exhausting the BER pathway and amplifying the generation of unrepaired DNA lesions. The XRCC1-depletion model we used in this study strongly indicates that accumulation of SSBs are sufficient to induce trans-differentiation because ROS and DSBs are undetectable in XRCC1 KD cells [12].

While providing a glimpse at the mechanisms driving CAF formation, this study also poses an important question about how DNA damage accumulation leads to genome-wide reprogramming. DNA damage has been observed to modulate a number of differentiation processes [31–33], although the mechanism(s) involved are unknown. Our proteomics analysis highlights major changes in proteins involved in chromatin remodelling and transcription. Thus, we speculate that generation of DNA repair defects and DNA damage on a whole-genome scale will likely lead to widespread chromatin reorganisation [34–37] and epigenetic modifications [38] and could be responsible for the transcriptional reprogramming observed in XRCC1 KD fibroblasts. Furthermore, we show that activation of stress-responsive transcription factors (e.g. ATF4) plays a crucial role in the trans-differentiation process.

Our bioinformatics analyses indicate that the CAF-like cells generated by depleting XRCC1 display a gene expression profile that is virtually indistinguishable from tumour stroma samples, whereby XRCC1 downregulation negatively correlates with expression of CAF markers. Intriguingly, downregulation of XRCC1 has been observed in a wide range of tumour types [39–42]. Low levels of XRCC1 expression have generally been associated with increased tumour aggressiveness and poorer outcome in triple negative breast cancer [41], reduced survival in gastric cancer [42] and unresponsiveness to radiotherapy in bladder cancer [43]. Moreover, low XRCC1 expression has recently been reported to impact prognosis in BRCA1-deficient breast cancers [44]. Our data suggest that XRCC1 expression levels in tumour stroma may be a crucial determinant for cancer progression and for the genesis of a tumour-promoting microenvironment. XRCC1 deficiency in the tumour stroma is likely, as demonstrated by our study, to act in synergy with ROS in the microenvironment and aggravate the accumulation of DNA breaks in order to amplify the pro-inflammatory response.

Targeting CAFs through either reversion of their phenotype, or selective elimination, is an appealing approach to cancer treatment. We demonstrate here that CAF-like cells generated by XRCC1 depletion are terminally committed to their “activated” status and therefore display an irreversible phenotype. Despite this, we devised a strategy that allows the selective elimination of a subset of CAFs showing BER deficiency. Our data indicate that midostaurin might be a promising drug to specifically neutralise BER-deficient stroma. Notably, midostaurin has recently been assigned a breakthrough therapy designation from the U.S. FDA for FLT3-mutated acute myeloid leukaemia (AML). The drug had previously been shown to induce apoptosis in keloid-derived fibroblasts [45]. Additionally, midostaurin has been shown to efficiently override the stroma-associated cytoprotection of AML cells, when combined with an AKT inhibitor [46]. During physiological wound healing, activated fibroblasts are eliminated by apoptosis [47]. Since CAFs are considered as their pathological equivalent, it is reasonable to assume that they could be eliminated in a similar fashion. This would suggest that BER-deficient CAF-like fibroblasts are in a pre-apoptotic state and should be sensitive to powerful apoptotic inducers such as staurosporine and midostaurin. It appears, therefore, that our data align with a growing body of evidence suggesting that midostaurin could have beneficial potential in multiple cancer types by virtue of its ability to target tumour stroma to apoptosis.

In summary, in this study we unveil a previously unrecognised concept, demonstrating that modulation of DNA repair in combination with inflammation stress could be a focal event during the formation of CAFs. These findings allowed us to identify midostaurin as a new drug targeting the tumour microenvironment. In combination with patient stratification based on BER status and capacity, our findings could constitute an extremely valuable approach to cancer treatment.

## MATERIALS AND METHODS

### Cell culture and drug treatments

Normal human fibroblasts TIG-1, AG16409 and WI38 were from the Coriell Institute Cell Repository. The non-small cell lung carcinoma cell line H1299 and A549 were obtained from the American Type Culture Collection (ATCC), while the bladder carcinoma cell line T24 was kindly provided by Dr Anne Kiltie (University of Oxford). All cell lines were cultured in DMEM (Life Technologies) supplemented with either 15% (for the fibroblast cell lines) or 10% FBS (for cancer cell lines) at 37° C in a humidified atmosphere with 5% CO_2_. Cells were routinely checked for mycoplasma. Recombinant human TGF-β1 (Peprotech) was used at the indicated concentration. The PLC inhibitor (edelfosine-Tocris), H_2_O_2_ (Sigma), the PDGF inhibitor (AG 1296-Millipore), staurosporine (Cell Guidance Systems), midostaurin (Sigma) and UCN-01 (Millipore) were used at the indicated concentrations.

### siRNA transfections

siRNA transfections were carried out using the Lipofectamine RNAiMAX reagent (Life Technologies) according to the manufacturer’s protocol. Unless otherwise indicated, cells were transfected with 30 nM siRNA and analysed 72 hours after transfection. siRNA oligonucleotides were obtained from Eurogentec; a detailed list of the sequences can be found in the SI Material and Methods. Control transfections were carried out using a non-targeting siRNA (Eurogentec, SR-CL000-005).

### Real-time PCR (qPCR)

Total RNA was extracted using the RNeasy kit (Qiagen) and cDNA was prepared using the SuperScript RT-PCR system (Life Technologies) as per manufacturer’s indications. Quantitative RT-PCR was performed using the Fast SYBR^®^ Green Master Mix (Applied Biosystems) according to the manufacturer’s protocol. Reactions were carried out using a 7500 Fast Real-Time PCR System (Applied Biosystems). The comparative CT method was applied for quantification of gene expression; *GAPDH* and *B2M* were used as endogenous controls. A list of the primers can be found in the SI Material and Methods.

### Comet assays

Cells were harvested by trypsinisation and analysed by alkaline comet assay as described elsewhere [12].

### *In vitro* ligation assays

Nuclear cell extracts were prepared as described previously [10]. Ligation assays were carried out using 1 mg of nuclear extract essentially as described in [48], with minor modifications. Briefly, reactions were performed in 50 mM Tris-HCl pH 7.5, 10 mM MgCl_2_, 10 mM DTT, 1 mM ATP at 37° C for the indicated time; the oligonucleotide substrate (50 nM) as previously described [48] was 5′-labelled with IRDye^®^800 (IDT). Reactions were halted with 96% formamide and 10 mM EDTA and analyzed by electrophoresis on a 20% denaturing polyacrylamide gel. The percentage of substrate converted to product was determined by using an Odyssey image analysis system (Li-Cor Biosciences).

### Western blot

Whole cell extracts for Western blot were prepared as described previously [49]. A list of the antibodies used can be found in the SI Material and Methods. Detection and quantification was carried out using an Odyssey image analysis system (Li-Cor Biosciences).

### 3D spheroids co-culture invasion assays

Cancer multicellular spheroids were generated by using the hanging drop method [50]. Briefly, cells were detached with 2 mmol/L EDTA, counted, re-suspended in DMEM supplemented with methylcellulose (20%; Sigma) and GFR Matrigel matrix (1%, Corning) and incubated as droplets (25 μL) containing 10^3^ cells for 48 hours to generate multicellular aggregates. To generate “hybrid” cell spheroids, equal numbers of H1299-GFP cells and fibroblasts (transfected with either control or XRCC1 siRNA) were mixed (10^3^ cells per droplet). For “hybrid” spheroids invasion, either cancer spheroids alone or “hybrid” aggregates were plated on Matrigel and cultured for two days in presence of medium conditioned by fibroblasts. Tumour cell invasion were apparent as projections from the spheroids after two days of co-culture. The invasion was scored by counting the invasive protrusions around the spheroids. At least 40 aggregates were counted per condition.

For network formation assay, cancer cell spheroids were washed and plated on Matrigel. 4 × 10^4^ fibroblasts (transfected with either control or XRCC1 siRNA) were added to the spheroids as a single-cell suspension. Network formation and cancer cell migration were evident after 24 hours. Images were captured by using a Nikon 10X/0.30 Ph1 objective.

### Immunostaining

Immunostaining was carried out following standard procedures. Briefly, cells were fixed with paraformaldehyde (4% in PBS for 15 minutes), or methanol:acetone (1:1) for α-SMA staining. Permeabilisation was carried out using Triton X-100 (0.2% in PBS for 10 minutes at 4° C) and cells were saturated with 5% bovine serum albumin (BSA) in PBS for 1 hour. Incubation with antibodies (see Supplementary Material) was carried out in 5% BSA-PBS supplemented with 0.01% Tween 20. Alexa Fluor 488- and Alexa Fluor 594-conjugated secondary antibodies (Life Technologies) were used for indirect detection of the antigens. Hoechst 33342 (Life Technologies) was used to visualise nuclei. For determination of 53BP1 foci and cell elongation, images were acquired using an IN Cell Analyzer 1000 Imaging System and data were analysed using the IN Cell Investigator Software (GE Healthcare Life Sciences). Cell elongation is represented as a ratio of the shorter axis of the cell divided by the longer axis. Data are expressed as distribution of the mean from six experimental replicates.

### Live cell imaging, wound healing assays and single-cell tracking

Cells were grown to 90% confluency before generation of a wound using a 200 µl tip. Cells were washed in PBS before adding fresh medium. Drug treatments were started 24 hours before wound scratching. Single-cell tracking commenced 5 hours after wound generation. The cells were observed under live microscopy using a Nikon Eclipse TE2000-E system for 20 hours capturing images every 30 minutes. Data were analysed using the ImageJ software and the MTrackJ plugin was used for single-cell tracking [51].

### Cancer cell growth assays

Cancer cell growth stimulation was assessed by incubating cells with medium conditioned by XRCC1 KD fibroblasts and by measuring their proliferation rate. In brief, TIG-1 cells were transfected with either a control or an XRCC1-targeting siRNA in serum-free medium. Conditioned medium was collected 72 hours after transfection and clarified by centrifugation at 1000 g for 5 minutes. T24 or H1299 cells were pre-seeded 48 hours before incubation with conditioned medium; incubation was then carried out for several days, measuring proliferation every 24 hours. Proliferation rate was measured by cell counting using Hoechst 33342 for T24 cells or using endogenous GFP fluorescence for H1299 cells. Fluorescence was quantified using a POLARstar Omega plate reader (BMG Labtech).

### Collagen gel contraction assays

Collagen gel contraction assays were performed essentially as described in [52]. Briefly, 72 hours after siRNA treatment, cells were trypsinised, counted and mixed with Cultrex^â^ Rat Collagen I (Trevigen). The mix was neutralised by addition of 1 M NaOH and transferred to 24 well plates. The collagen gels were allowed to solidify for 30 minutes at 37° C and 600 µl of culture medium was added to each well. Gel plugs were gently detached from the wells and plates were incubated for 24 hours at 37° C. To measure gel contraction, plugs were imaged using a ChemiDoc™ system (BioRad) and gel areas were quantified using ImageJ software. Contractility was calculated by measuring the difference in area between the collagen plug and the entire well.

### Boyden chamber invasion assays

Cancer cells (10^5^) were cultured in triplicate in the top wells of Transwell Matrigel chambers (Corning) and allowed to invade towards fibroblasts (transfected with either control or XRCC1 siRNA) cultured in the bottom wells in conditioned medium supplemented with 10% FBS. Eighteen hours later invading cells were stained with Richard-Allan Scientific™ Three-Step Stain (Thermo Fisher Scientific), imaged and counted manually using Adobe Photoshop software.

### Viability and apoptosis assays

Cell viability was assessed using resazurin (Sigma). Apoptosis induction was measured by using the Apo-ONE^®^ Homogeneous Caspase-3/7 Assay (Promega), according to the manufacturer’s protocol. Specificity of the signal was confirmed by co-incubating cells with the relevant drug and the pan-caspase inhibitor Z-VAD(OMe)-FMK (Insight Biotechnology) during apoptosis induction.

### Statistical analyses

Statistical analyses were performed by using the two-tailed Student’s *t*-test using either Microsoft Excel or SPSS (IBM). Sample size is indicated for each experiment.

## SUPPLEMENTARY MATERIALS FIGURES AND TABLES









## References

[1] Hanahan D, Coussens LM (2012). Accessories to the crime: functions of cells recruited to the tumor microenvironment. Cancer Cell.

[2] Kalluri R (2016). The biology and function of fibroblasts in cancer. Nat Rev Cancer.

[3] Sugimoto H, Mundel TM, Kieran MW, Kalluri R (2006). Identification of fibroblast heterogeneity in the tumor microenvironment. Cancer Biol Ther.

[4] Erez N, Truitt M, Olson P, Arron ST, Hanahan D (2010). Cancer-Associated Fibroblasts Are Activated in Incipient Neoplasia to Orchestrate Tumor-Promoting Inflammation in an NF-kappaB-Dependent Manner. Cancer Cell.

[5] Calon A, Tauriello DV, Batlle E (2014). TGF-beta in CAF-mediated tumor growth and metastasis. Semin Cancer Biol.

[6] Costa A, Scholer-Dahirel A, Mechta-Grigoriou F (2014). The role of reactive oxygen species and metabolism on cancer cells and their microenvironment. Semin Cancer Biol.

[7] van Loon B, Markkanen E, Hubscher U (2010). Oxygen as a friend and enemy: How to combat the mutational potential of 8-oxo-guanine. DNA Repair (Amst).

[8] Bohr VA, Dianov GL (1999). Oxidative DNA damage processing in nuclear and mitochondrial DNA.. Biochimie.

[9] Dianov GL, Hubscher U (2013). Mammalian base excision repair: the forgotten archangel. Nucleic Acids Res.

[10] Markkanen E, Fischer R, Ledentcova M, Kessler BM, Dianov GL (2015). Cells deficient in base-excision repair reveal cancer hallmarks originating from adjustments to genetic instability. Nucleic Acids Res.

[11] Caldecott KW (2003). XRCC1 and DNA strand break repair. DNA Repair (Amst).

[12] Khoronenkova SV, Dianov GL (2015). ATM prevents DSB formation by coordinating SSB repair and cell cycle progression. Proc Natl Acad Sci U S A.

[13] Kustatscher G, Wills KL, Furlan C, Rappsilber J (2014). Chromatin enrichment for proteomics. Nat Protoc.

[14] Kalluri R, Zeisberg M (2006). Fibroblasts in cancer. Nat Rev Cancer.

[15] Hinz B, Phan SH, Thannickal VJ, Galli A, Bochaton-Piallat ML, Gabbiani G (2007). The myofibroblast: one function, multiple origins. Am J Pathol.

[16] Goicoechea SM, Garcia-Mata R, Staub J, Valdivia A, Sharek L, McCulloch CG, Hwang RF, Urrutia R, Yeh JJ, Kim HJ, Otey CA (2014). Palladin promotes invasion of pancreatic cancer cells by enhancing invadopodia formation in cancer-associated fibroblasts. Oncogene.

[17] Follonier L, Schaub S, Meister JJ, Hinz B (2008). Myofibroblast communication is controlled by intercellular mechanical coupling. J Cell Sci.

[18] Romero JR, Rivera A, Lanca V, Bicho MD, Conlin PR, Ricupero DA (2005). Na+/Ca2+ exchanger activity modulates connective tissue growth factor mRNA expression in transforming growth factor beta1- and Des-Arg10-kallidin-stimulated myofibroblasts. J Biol Chem.

[19] Bonavita E, Gentile S, Rubino M, Maina V, Papait R, Kunderfranco P, Greco C, Feruglio F, Molgora M, Laface I, Tartari S, Doni A, Pasqualini F (2015). PTX3 is an extrinsic oncosuppressor regulating complement-dependent inflammation in cancer. Cell.

[20] Karnoub AE, Dash AB, Vo AP, Sullivan A, Brooks MW, Bell GW, Richardson AL, Polyak K, Tubo R, Weinberg RA (2007). Mesenchymal stem cells within tumour stroma promote breast cancer metastasis. Nature.

[21] Finak G, Bertos N, Pepin F, Sadekova S, Souleimanova M, Zhao H, Chen H, Omeroglu G, Meterissian S, Omeroglu A, Hallett M, Park M (2008). Stromal gene expression predicts clinical outcome in breast cancer. Nat Med.

[22] Garnett MJ, Edelman EJ, Heidorn SJ, Greenman CD, Dastur A, Lau KW, Greninger P, Thompson IR, Luo X, Soares J, Liu Q, Iorio F, Surdez D (2012). Systematic identification of genomic markers of drug sensitivity in cancer cells. Nature.

[23] Griner EM, Kazanietz MG (2007). Protein kinase C and other diacylglycerol effectors in cancer. Nat Rev Cancer.

[24] Prakash J (2016). Cancer-Associated Fibroblasts: Perspectives in Cancer Therapy. Trends Cancer.

[25] Ohlund D, Elyada E, Tuveson D (2014). Fibroblast heterogeneity in the cancer wound. J Exp Med.

[26] Darby IA, Hewitson TD (2007). Fibroblast differentiation in wound healing and fibrosis. Int Rev Cytol.

[27] Grivennikov SI, Greten FR, Karin M (2010). Immunity, inflammation, and cancer. Cell.

[28] Krstic J, Trivanovic D, Mojsilovic S, Santibanez JF (2015). Transforming Growth Factor-Beta and Oxidative Stress Interplay: Implications in Tumorigenesis and Cancer Progression. Oxid Med Cell Longev.

[29] Hubackova S, Krejcikova K, Bartek J, Hodny Z (2012). IL1- and TGFbeta-Nox4 signaling, oxidative stress and DNA damage response are shared features of replicative, oncogene-induced, and drug-induced paracrine ‘bystander senescence’. Aging (Albany NY).

[30] Jain M, Rivera S, Monclus EA, Synenki L, Zirk A, Eisenbart J, Feghali-Bostwick C, Mutlu GM, Budinger GR, Chandel NS (2013). Mitochondrial reactive oxygen species regulate transforming growth factor-beta signaling. J Biol Chem.

[31] Sherman MH, Bassing CH, Teitell MA (2011). Regulation of cell differentiation by the DNA damage response. Trends Cell Biol.

[32] Santos MA, Faryabi RB, Ergen AV, Day AM, Malhowski A, Canela A, Onozawa M, Lee JE, Callen E, Gutierrez-Martinez P, Chen HT, Wong N, Finkel N (2014). DNA-damage-induced differentiation of leukaemic cells as an anti-cancer barrier. Nature.

[33] Wang H, Bierie B, Li AG, Pathania S, Toomire K, Dimitrov SD, Liu B, Gelman R, Giobbie-Hurder A, Feunteun J, Polyak K, Livingston DM (2016). BRCA1/FANCD2/BRG1-Driven DNA Repair Stabilizes the Differentiation State of Human Mammary Epithelial Cells. Mol Cell.

[34] Mikhed Y, Gorlach A, Knaus UG, Daiber A (2015). Redox regulation of genome stability by effects on gene expression, epigenetic pathways and DNA damage/repair. Redox Biol.

[35] O’Hagan HM (2014). Chromatin modifications during repair of environmental exposure-induced DNA damage: a potential mechanism for stable epigenetic alterations. Environ Mol Mutagen.

[36] Polo SE (2015). Reshaping chromatin after DNA damage: the choreography of histone proteins. J Mol Biol.

[37] Schick S, Fournier D, Thakurela S, Sahu SK, Garding A, Tiwari VK (2015). Dynamics of chromatin accessibility and epigenetic state in response to UV damage. J Cell Sci.

[38] Fleming AM, Ding Y, Burrows CJ (2017). Oxidative DNA damage is epigenetic by regulating gene transcription via base excision repair. Proc Natl Acad Sci U S A.

[39] Bajpai D, Banerjee A, Pathak S, Jain SK, Singh N (2013). Decreased expression of DNA repair genes (XRCC1, ERCC1, ERCC2, and ERCC4) in squamous intraepithelial lesion and invasive squamous cell carcinoma of the cervix. Mol Cell Biochem.

[40] Blomquist T, Crawford EL, Mullins D, Yoon Y, Hernandez DA, Khuder S, Ruppel PL, Peters E, Oldfield DJ, Austermiller B, Anders JC, Willey JC (2009). Pattern of antioxidant and DNA repair gene expression in normal airway epithelium associated with lung cancer diagnosis. Cancer Res.

[41] Sultana R, Abdel-Fatah T, Abbotts R, Hawkes C, Albarakati N, Seedhouse C, Ball G, Chan S, Rakha EA, Ellis IO, Madhusudan S (2013). Targeting XRCC1 deficiency in breast cancer for personalized therapy. Cancer Res.

[42] Wang S, Wu X, Chen Y, Zhang J, Ding J, Zhou Y, He S, Tan Y, Qiang F, Bai J, Zeng J, Gong Z, Li A (2012). Prognostic and predictive role of JWA and XRCC1 expressions in gastric cancer. Clin Cancer Res.

[43] Sak SC, Harnden P, Johnston CF, Paul AB, Kiltie AE (2005). APE1 and XRCC1 protein expression levels predict cancer-specific survival following radical radiotherapy in bladder cancer. Clin Cancer Res.

[44] Albarakati N, Abdel-Fatah TM, Doherty R, Russell R, Agarwal D, Moseley P, Perry C, Arora A, Alsubhi N, Seedhouse C, Rakha EA, Green A, Ball G (2015). Targeting BRCA1-BER deficient breast cancer by ATM or DNA-PKcs blockade either alone or in combination with cisplatin for personalized therapy. Mol Oncol.

[45] Nakazono-Kusaba A, Takahashi-Yanaga F, Miwa Y, Morimoto S, Furue M, Sasaguri T (2004). PKC412 induces apoptosis through a caspase-dependent mechanism in human keloid-derived fibroblasts. Eur J Pharmacol.

[46] Weisberg E, Liu Q, Zhang X, Nelson E, Sattler M, Liu F, Nicolais M, Zhang J, Mitsiades C, Smith RW, Stone R, Galinsky I, Nonami A (2013). Selective Akt inhibitors synergize with tyrosine kinase inhibitors and effectively override stroma-associated cytoprotection of mutant FLT3-positive AML cells. PLoS One.

[47] Darby IA, Zakuan N, Billet F, Desmouliere A (2015). The myofibroblast, a key cell in normal and pathological tissue repair. Cell Mol Life Sci.

[48] McNeill DR, Narayana A, Wong HK, Wilson DM (2004). Inhibition of Ape1 nuclease activity by lead, iron, and cadmium. Environ Health Perspect.

[49] Orlando G, Khoronenkova SV, Dianova II, Parsons JL, Dianov GL (2014). ARF induction in response to DNA strand breaks is regulated by PARP1. Nucleic Acids Res.

[50] Foty R (2011). A simple hanging drop cell culture protocol for generation of 3D spheroids. J Vis Exp.

[51] Meijering E, Dzyubachyk O, Smal I (2012). Methods for cell and particle tracking. Methods Enzymol.

[52] Ngo P, Ramalingam P, Phillips JA, Furuta GT (2006). Collagen gel contraction assay. Methods Mol Biol.

