# Supplementary material for: Persistent DNA strand breaks induce a CAF-like phenotype in normal fibroblasts
Source: Oncotarget. 2018 Feb 7;9(17):13666–81. doi: 10.18632/oncotarget.24446 (PMC5862606; doi:10.18632/oncotarget.24446)
Supplement: Supplementary file 1 [file oncotarget-09-13666-s001.pdf]

Persistent DNA strand breaks induce a caf-like phenotype in normal fibroblasts

SUPPLEMENTARY MATERIALS

Experimental procedures

siRNA sequences

Target	Sequence (5′ to 3′)
XRCC1	AGGGAAGAGGAAGUUGGAU
XRCC1 #2	GCUUGAGUUUUGUACGGUU
XRCC1 #3	GGAAGAUAUAGACAUUGAG
ATF4 #1*	GCCUAGGUCUCUUAGAUGA
ATF4 #2*	CUGCUUACGUUGCCAUGAU
p21	GAUGGAACUUCGACUUUGU

*Used together as a pool.

qPCR primer sequences

Target	Forward primer (5′ to 3′)	Reverse primer (5′ to 3′)
ALDH2	CCTCTCCAGTGGACGGATT	CGAGGTCTTCTGCAACCAG
ACTA2 (α-SMA)	TCAATGTCCCAGCCATGTAT	CAGCACGATGCCAGTTGT
ATF4	GGGACAGATTGGATGTTGGAGA	ACCCAACAGGGCATCCAAGT
B2M	ATGTCTCGCTCCGTGGCCTTA	ATCTTGGGCTGTGACAAAGTC
BRE	CCTGGAAAAGCTGGGATATG	TTTTGAGGACACTGAAGCCA
CADM1	CAACCTCTCCCTCGATCACT	GGCTTCTGCTGTTGCTCTTC
CAV1	ACAGCCCAGGGAAACCTC	GATGGGAACGGTGTAGAGATG
CLIC4	CACGTAAATTTCTGGATGGCAATG	ATCACTGGGACAGGTATTGGTGAAC
CTGF	TTGGCCCAGACCCAACTA	GCAGGAGGCGTTGTCATT
COL1A1	TGGTCCACTTGCTTGAAGAC	ACAGATTTGGGAAGGAGTGG
COL1A2	AGGAGCTCCAAGGACAAGAA	ATGAAGGCAAGTTGGGTAGC
DFNA5	CACTTCTGCATCGTCGTGAT	AGGAAGCAGGAGGTGGATTT
FAP	TCAGTGTGAGTGCTCTCATTGTAT	GCTGTGCTTGCCTTATTGGT
GAPDH	AGCCACATCGCTCAGACAC	GCCCAATACGACCAAATCC
IGFBP7	GCTCAAGTACACCTGGGCAC	CATCACCCAGGTCAGCAAG
IL-6	GACAGCCACTCACCTCTTCA	CCTCTTTGCTGCTTTCACAC
IRF2BP2	CCGACTTCACCTTCTGGTTC	AGGTTGTTGGGTTTCGAGG
MMP2	GGAATGCCATCCCCGATAAC	CAGCCTAGCCAGCCAGTCGGATTT
PALLD	AACCGAGCAGGACAGAAC	TGGTGGCACTCCCAATAC
PDGF-D	TTCCTTTCACCTGGATGGTC	GTCGGGACACTTCTGCAAC
PDGFRA	CAATCCAAAGATATCCAGCTC	ACCAAGTCAGGCCCCATTTAC
PDGFRB	CAGGAGAGACAGCAACAGCA	AACTGTGCCCACACCAGAAG
PDLIM5	ATTCTTACATTTGCCTGGGCT	ACAGTGTGTCACTGGTTGGC

PML	ATGAAGTGCTACGCCTCGGAC	CCCCTGGGTGATGCAAGAGCT
PTX3	GCATCTCCTTGCGATTCTGTT	CATTCCGAGTGCTCCTGACC
RBM10	TGGCTGGGAAGTGAAACGGA	GGATGTTGAGGGAGTGCTGA
SF3B2	CTGGCAACACAGTGAAGAGC	GGAATGGAGACCCCTGAACT
SPARC	GAAAGAAGATCCAGGCCCTC	CTTCAGACTGCCCGGAGA
TACC1	TTCAACCACTTCTGCTACAAGC	TGTGATCCATCACTCGGATT
TOP2A	TGCCAATGTAGTTTGTTTCTTTG	GCCCTCAAGAAGATGGTGTG
TPM1	CGGTCGGCATCTTCAGCAATG	GAGAGTGAGAGAGGCATGAAAGTC
UHRF1	GCAGAGGCTGTTCTACAGGG	GTGTCGGAGAGCTCGGAGT
WNT5A	TCCTCTCGCCCATGGAATTA	CATTGCACTTCCAGCCATCC
ZC3H7A	CACTGTTGCAGTTTTTCCCA	AGCCAGTCAGTCAAACAAGGA

Antibodies

Target	Antibody
XRCC1	MS-1393-P0–Thermo Scientific
α-SMA	ab5694–Abcam
PALLD	NBP1-25959–Novus Biologicals
β-Actin	ab6276–Abcam
α-Tubulin	T6199–Sigma
53BP1	A300-272A–Bethyl Laboratories
Vinculin	sc-73614–Santa Cruz

Supplementary Table 1: Protein abundance data from the SILAC analysis. Data obtained from the SILAC analysis on XRCC1-depleted cells are reported as fold change in protein abundance relative to control cells. N.I.: not identified. See Supplementary_ Table_1

Supplementary Table 2: List of CAF markers identified upon XRCC1 depletion

Marker	Status in CAF	Fold change upon XRCC1 depletion (SILAC)			Transcription (qPCR)	Reference
		ChEP	Nuc.	Cyt.		
PALLD	Up	1.56	2.09	1.98	Up	[1]
ACTA2/α-SMA	Up	0.28	1.71	1.42	Up	[2]
TPM1	Up	1.65	2.41	0.65	Up	[3]
TPM2	Up	1.51	1.90	1.16	N.T.	[4]
CNN2	Up	1.43	1.40	1.28	N.T.	[4]
MYH9	Up	1.53	1.13	1.13	N.T.	[5]
MYL9	Up	N.I.	1.69	1.41	N.T.	[5]
CALD1	Up	1.91	1.74	1.54	N.T.	[6]
PDLIM5	Up	N.I.	N.I.	2.30	No change	[7]
IGFBP7	Up	N.I.	2.66	2.93	Up	[8]
MMP2	Up	N.I.	2.59	2.11	Up	[9]
SPARC	Up	N.I.	2.15	1.52	Up	[4]
COL1A1	Up	1.39	3.35	5.75	Up	[4]
PDGFRA	Up	N.I.	1.70	1.26	Up	[2]
PDGFRB	Up	N.I.	1.55	1.56	Up	[2]
ICAM1	Up	N.I.	N.I.	2.72	N.T.	[10]
FAP	Up	N.I.	0.66	1.21	Up	[2]
FSP1	Up	N.I.	0.45	N.I.	N.T.	[2]
CAV1	Down	N.I.	1.06	1.15	Down	[4]
GSTP1	Down	0.64	1.19	N.I.	N.T.	[11]
PML	Up	10.15	N.I.	0.95	Up	[12]

List of known markers for CAFs identified by our analysis. The status of every marker as generally detected in CAFs is reported (Up: upregulated, Down: downregulated), together with their fold change as detected in the SILAC proteomics analyses. For a subset of the markers transcription analyses were also carried out. N.I.: not identified, N.T.: not tested.

Supplementary Table 3: Correlation data obtained from the SEEK database. Correlation data obtained from the SEEK database using the indicated datasets. The database was searched against XRCC1 expression as a whole “alldatabase”, as collection of stromal cells “stromalcells”, or as a source of data on fibroblasts “fibroblast”. Each search generated a co-expression score indicating the correlation with XRCC1 expression. For each search the datasets employed have been reported in the table. Supplementary Table 3 is provided as an Excel spreadsheet. See Supplementary_Table_3

Supplementary Table 4: Gene Ontology enrichment analysis. Gene Ontology enrichment analysis carried out using the GOrilla software and analysing the 500 top/bottom hits obtained from the SEEK database as described in Supplementary Table 3. We focussed on the fibroblast dataset for this analysis. For each search we report the GO terms, along with their fold enrichment and relative *p*-value. Supplementary Table 4 is provided as an Excel spreadsheet. See Supplementary_Table_4

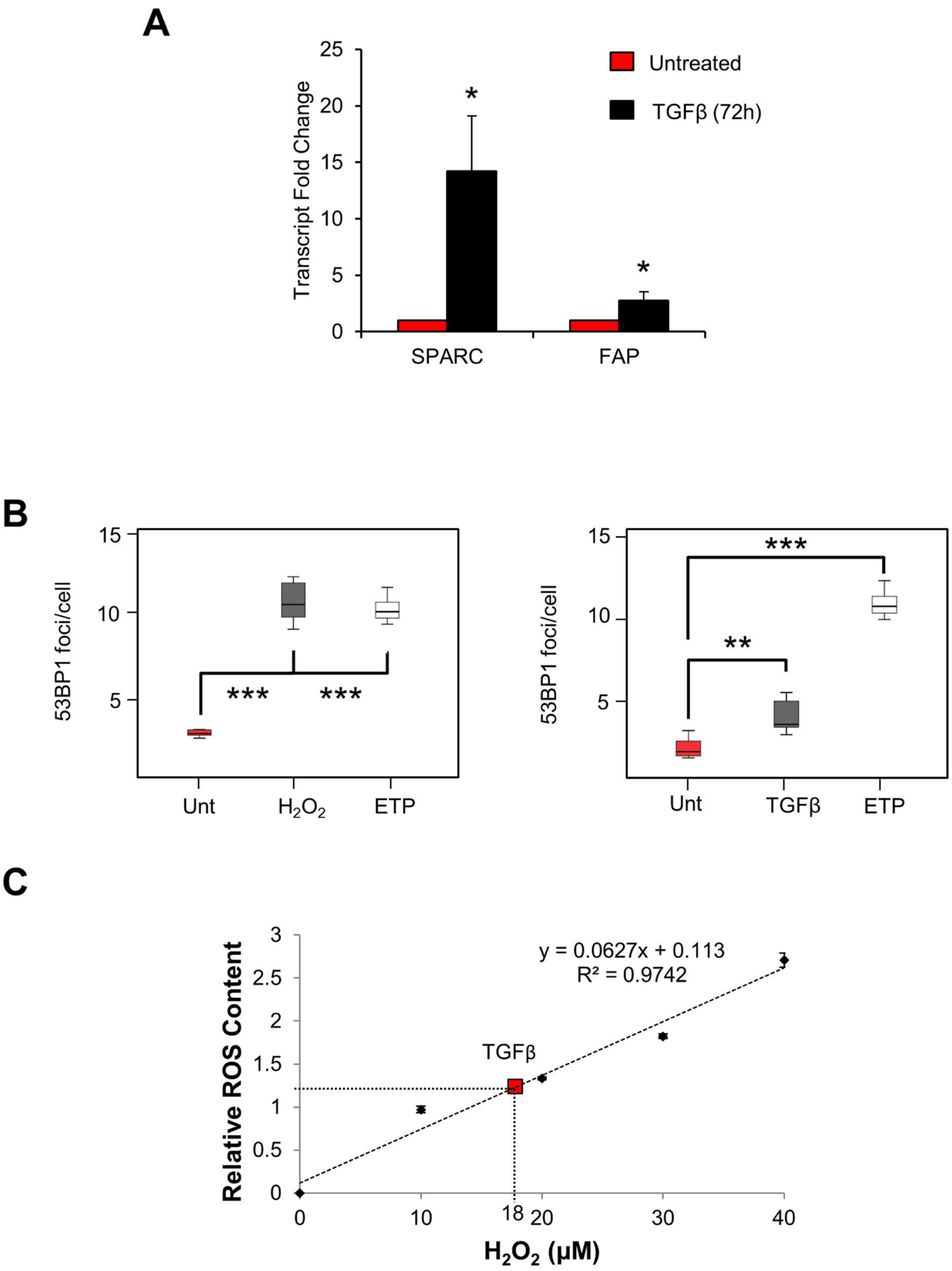

Supplementary Figure 1: Persistent exposure of fibroblasts to ROS or TGFβ leads to accumulation of DNA strand breaks. (**A**) Effect of TGFβ on the expression of SPARC and FAP. TIG-1 fibroblasts were treated with TGFβ (10 ng/ml) for 72 hours. Gene expression was analysed by qPCR. (**B**) Effect of TGFβ (10 ng/ml) and H2O2 (125 μM) on the levels of DNA damage. TIG-1 fibroblasts were treated as indicated; drugs were administered every 24 h for a total of 72 h using the indicated concentrations. DNA damage was assessed by scoring 53BP1 foci formation using high-throughput immuno-fluorescence. Etoposide (10 μM, for 6 h – ETP) was used as a positive control. (**C**) Effect of TGFβ (10 ng/ml) and H2O2 (10–40 μM) on the levels of intracellular ROS. Cells were treated as in (B) and samples were analysed by FACS after 72 h. Results are presented as mean ± SD of three independent experiments. $^{*}p < 0.05$; $^{**}p < 0.01$; $^{***}p < 0.001$.

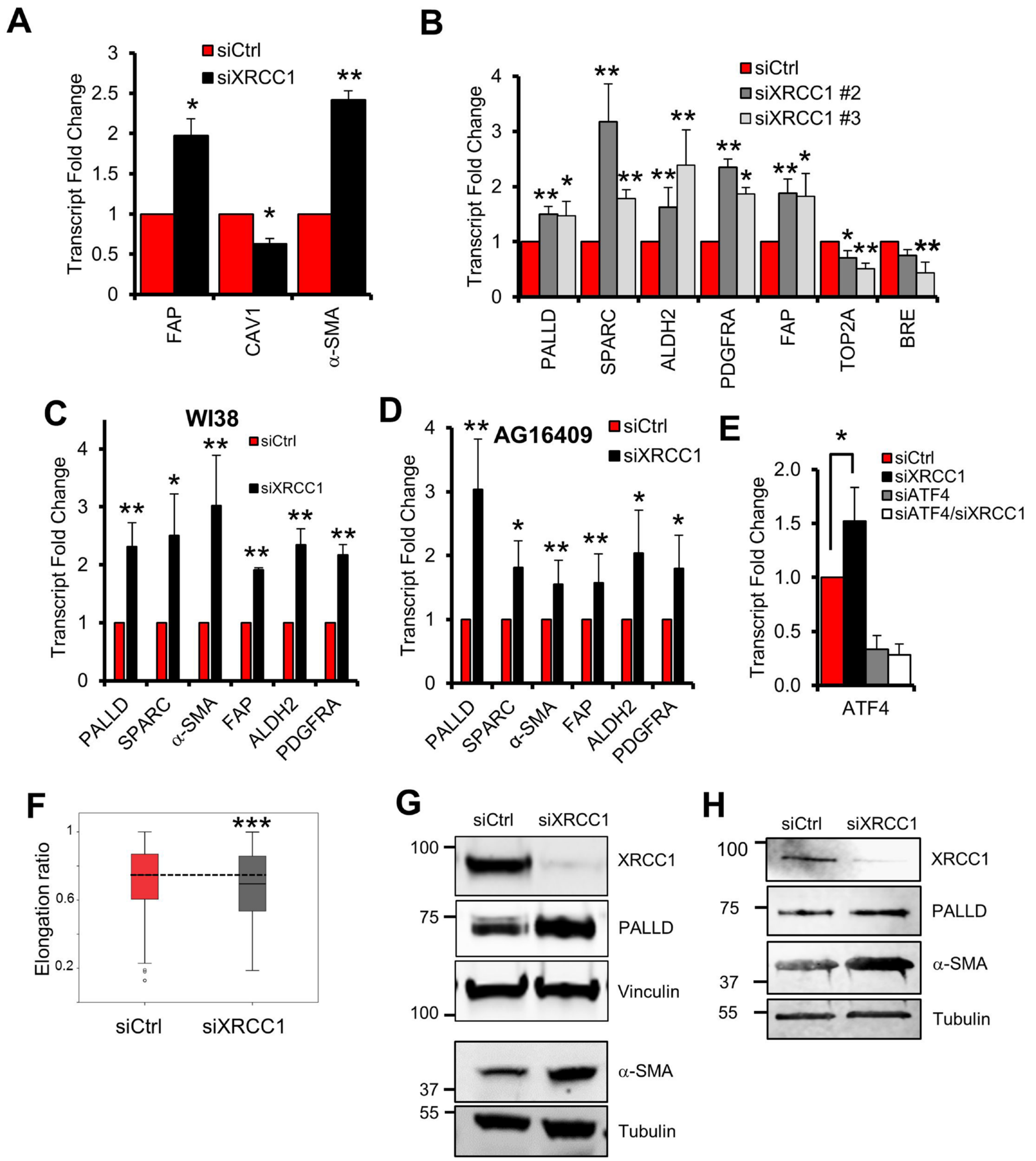

Supplementary Figure 2: XRCC1 depletion leads to reprogramming of normal fibroblasts into CAF-like cells. (**A**) Effect of XRCC1 depletion on FAP, CAV1 and α-SMA expression levels. TIG-1 cells were transfected with the indicated siRNA and gene expression was analysed 72 h later by qPCR. (**B**) Effect of XRCC1 depletion using different siRNA sequences on expression of CAF markers. TIG-1 cells were treated as in (A) using the indicated siRNA sequences. Gene expression was analysed by qPCR. (**C**–**D**) Effect of XRCC1 depletion on the expression of CAF markers in WI38 (C) or AG16409 (D) cells. Cells were treated as in (A) using the indicated siRNAs. Gene expression was analysed by qPCR. (**E**) XRCC1 depletion increases ATF4 expression. TIG-1 cells were transfected with the indicated siRNA and gene expression was analysed 72 h later by qPCR. (**F**) XRCC1 KD WI38 fibroblasts show an elongated shape. Cell elongation ratio was assessed by IN Cell high-throughput imagery, as described in Materials and methods. Statistical significance was evaluated by a non-parametric Mann-Whitney *U* Test. (**G**) XRCC1 depletion leads to increased expression of PALLD and α-SMA in WI38 cells. WI38 fibroblasts were treated as in (C) and analysed by western blotting. (**H**) XRCC1 depletion leads to increased expression of PALLD and α-SMA in AG16409 cells. AG16409 fibroblasts were treated as in (D) and analysed by western blotting. Data are expressed as mean ± SD of at least three independent experiments $^{*}p < 0.05$; $^{**}p < 0.01$; $^{***}p < 0.001$.

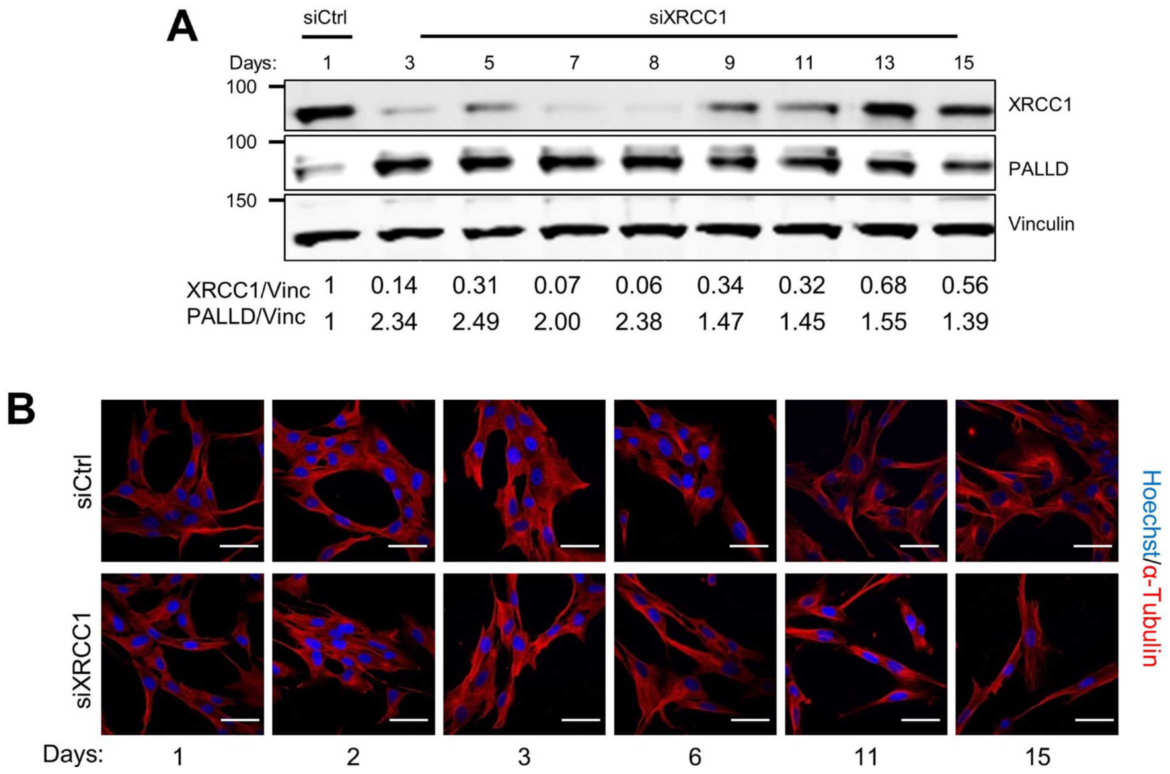

Supplementary Figure 3: XRCC1 KD fibroblasts show no reversion of the CAF-like phenotype after 15 days. (**A**) Persistency of PALLD upregulation upon XRCC1 depletion. TIG-1 fibroblasts were transfected twice with an XRCC1-targeting siRNA at day 0 and day 4, respectively. Cells were harvested at the indicated time points after transfection and analysed by Western blot. (**B**) Persistency of cytoskeleton contraction upon XRCC1 depletion. TIG-1 fibroblasts were treated as in (A). Cells were fixed at the indicated time points and stained for α-tubulin. Nuclei were visualised with Hoechst. Scale bars: 50 µm.

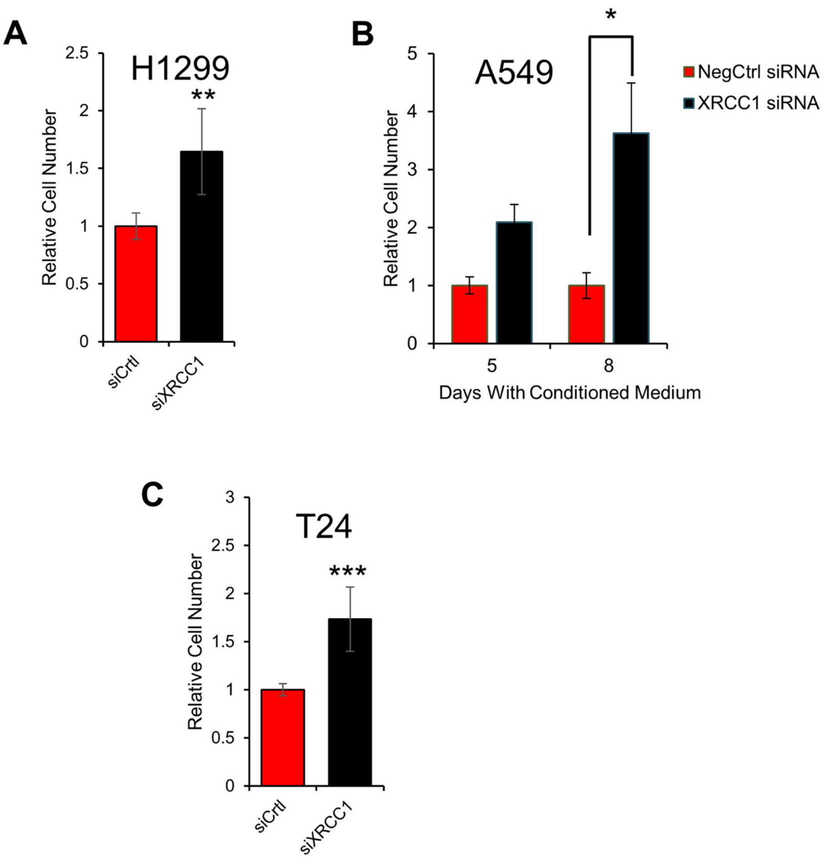

Supplementary Figure 4: Secretion from XRCC1 KD fibroblasts promotes growth of cancer cells. (**A**–**B**) Stimulation of proliferation of H1299 (B) or T24 (C) cells by medium conditioned by XRCC1 KD fibroblasts (WI38 cells). WI38 fibroblasts were treated with the indicated siRNAs for 72 h, conditioned medium was then collected and used to feed cancer cells for five days. (**C**) Stimulation of proliferation of A549 cells by medium conditioned by XRCC1 KD fibroblasts. TIG-1 fibroblasts were treated with the indicated siRNAs for 72 h, conditioned medium was then collected and used to feed cancer cells for five or eight days. Data are expressed as mean ± SD of at least three independent experiments $^{*}p < 0.05$; $^{***}p < 0.001$.

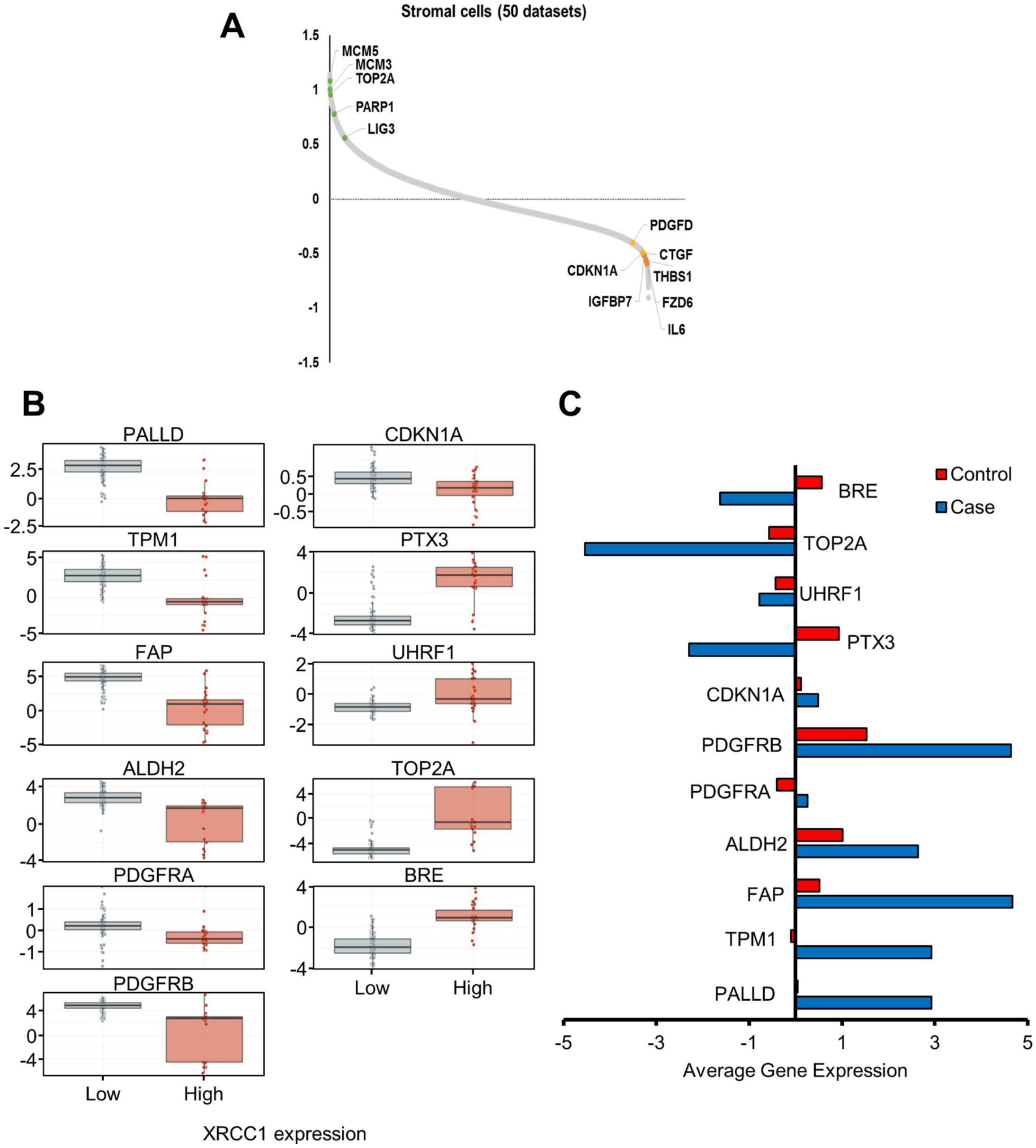

Supplementary Figure 5: Analysis of clinical stroma samples supports the link between BER gene expression and CAF markers. (**A**) Plot showing the correlation between XRCC1 expression levels and the rest of the genome in 50 stromal cell datasets from the SEEK database. BER genes and genes associated with replication (green dots) positively correlate with XRCC1 expression, whereas CAF markers (yellow and red) negatively correlate with XRCC1 expression. Correlation is expressed as Z-score. A complete list of genes correlating with XRCC1 expression can be found in Table S3. (**B**) Boxplots showing the gene expression distribution for the genes analysed in Figure 5D. For all the boxplot the difference shown is statistically significant ($p < 0.01$). (**C**) Histogram showing the average expression of the indicated genes of interest in case vs. control samples in the analysis reported in Figure 5B.

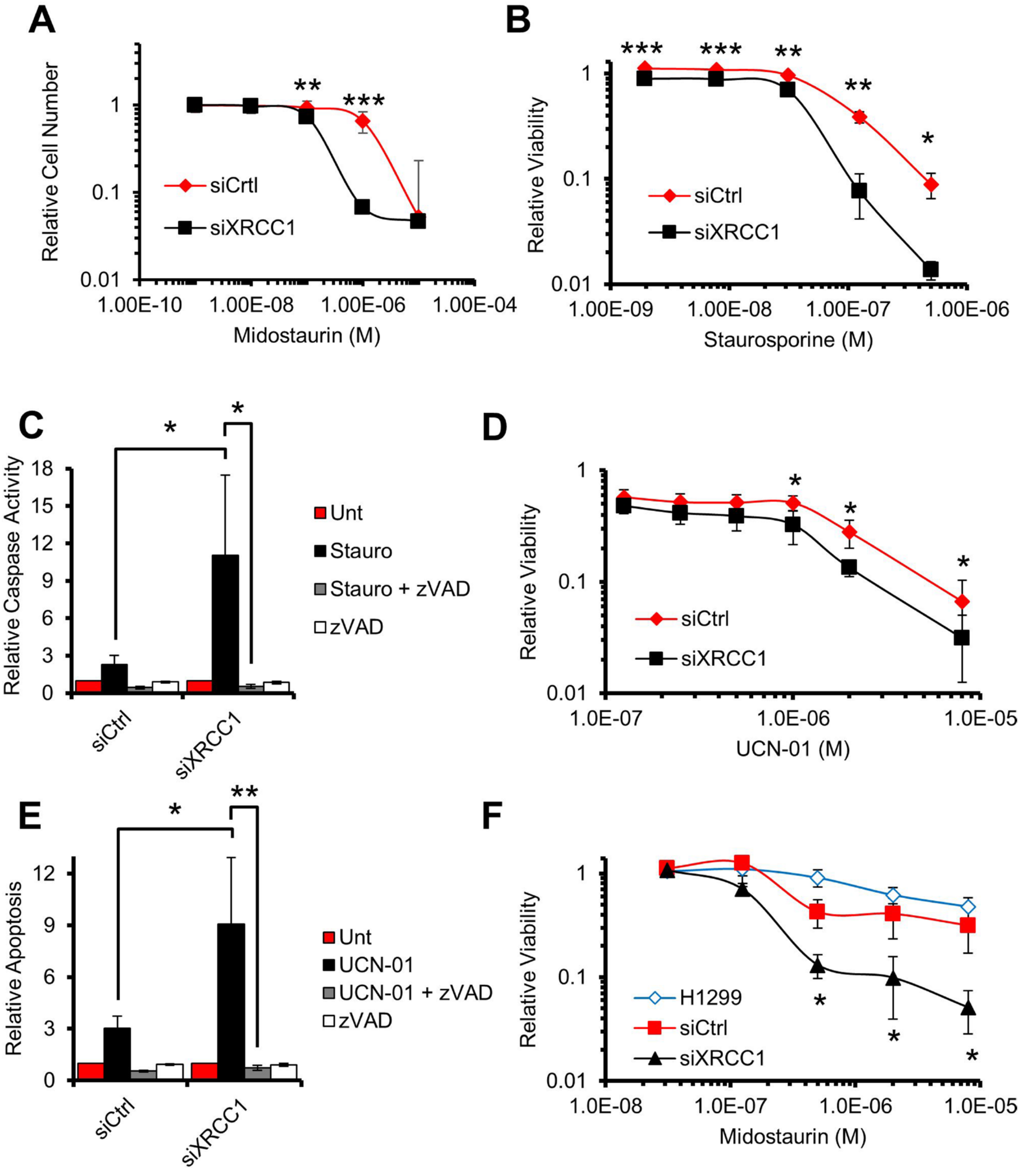

Supplementary Figure 6: CAF-like cells generated by BER depletion are hyper-sensitive to staurosporine derivatives. (**A**) Sensitivity of XRCC1 KD fibroblasts to midostaurin. WI38 fibroblasts were treated with the indicated siRNA for 48 h before exposure to increasing concentrations of midostaurin for 72 h. Cell number was assessed using Hoechst. (**B**, **D**) Sensitivity of XRCC1 KD fibroblasts to staurosporine or UCN-01. TIG-1 fibroblasts were treated with the indicated siRNA for 48 h before exposure to increasing concentrations of staurosporine (A) or UCN-01 (C) for 24 h. Cell viability was assessed using resazurin. (**C**, **E**) Increased apoptosis in XRCC1 KD fibroblasts upon exposure to staurosporine or UCN-01. TIG-1 fibroblasts were treated as in (A). Caspase activity was assessed after 24 h, upon incubation with staurosporine (125 nM (B)) UCN-01 (1.5 µM (D)). Specificity of caspase activation was confirmed by co-incubation of UCN-01 with the pan-caspase inhibitor, zVAD. (F) Sensitivity of H1299 cancer cells to midostaurin. H1299 were exposed to increasing concentrations of midostaurin for 72 h and their sensitivity to the treatment was compared to TIG-1 fibroblasts (see Figure 6). Cell viability was assessed using resazurin. Results are presented as mean ± SD of at least three independent experiments. $^{*}p < 0.05$; $^{**}p < 0.01$.

REFERENCES

1. Goicoechea SM, Garcia-Mata R, Staub J, Valdivia A, Sharek L, McCulloch CG, Hwang RF, Urrutia R, Yeh JJ, Kim HJ, Otey CA. Palladin promotes invasion of pancreatic cancer cells by enhancing invadopodia formation in cancer-associated fibroblasts. Oncogene. 2014; 33:1265–73.
2. Calon A, Tauriello DV, Batlle E. TGF-beta in CAF-mediated tumor growth and metastasis. Semin Cancer Biol. 2014; 25:15–22.
3. Pavlides S, Tsirigos A, Vera I, Flomenberg N, Frank PG, Casimiro MC, Wang C, Pestell RG, Martinez-Outschoorn UE, Howell A, Sotgia F, Lisanti MP. Transcriptional evidence for the "Reverse Warburg Effect" in human breast cancer tumor stroma and metastasis: similarities with oxidative stress, inflammation, Alzheimer's disease, and "Neuron-Glia Metabolic Coupling". Aging (Albany NY). 2010; 2:185–99. https://doi.org/10.18632/aging.100134.
4. Pavlides S, Whitaker-Menezes D, Castello-Cros R, Flomenberg N, Witkiewicz AK, Frank PG, Casimiro MC, Wang C, Fortina P, Addya S, Pestell RG, Martinez-Outschoorn UE, Sotgia F, et al. The reverse Warburg effect: aerobic glycolysis in cancer associated fibroblasts and the tumor stroma. Cell Cycle. 2009; 8:3984–4001.
5. Calvo F, Ege N, Grande-Garcia A, Hooper S, Jenkins RP, Chaudhry SI, Harrington K, Williamson P, Moeendarbary E, Charras G, Sahai E. Mechanotransduction and YAP-dependent matrix remodelling is required for the generation and maintenance of cancer-associated fibroblasts. Nat Cell Biol. 2013; 15:637–46.
6. Calon A, Lonardo E, Berenguer-Llergo A, Espinet E, Hernando-Momblona X, Iglesias M, Sevillano M, Palomo-Ponce S, Tauriello DV, Byrom D, Cortina C, Morral C, Barcelo C, et al. Stromal gene expression defines poor-prognosis subtypes in colorectal cancer. Nat Genet. 2015; 47:320–9.
7. Edlund K, Lindskog C, Saito A, Berglund A, Ponten F, Goransson-Kultima H, Isaksson A, Jirstrom K, Planck M, Johansson L, Lambe M, Holmberg L, Nyberg F, et al. CD99 is a novel prognostic stromal marker in non-small cell lung cancer. Int J Cancer. 2012; 131:2264–73.
8. Rupp C, Scherzer M, Rudisch A, Unger C, Haslinger C, Schweifer N, Artaker M, Nivarthi H, Moriggl R, Hengstschlager M, Kerjaschki D, Sommergruber W, Dolznig H, et al. IGFBP7, a novel tumor stroma marker, with growth-promoting effects in colon cancer through a paracrine tumor-stroma interaction. Oncogene. 2015; 34:815–25.
9. Yu B, Chen X, Li J, Qu Y, Su L, Peng Y, Huang J, Yan J, Yu Y, Gu Q, Zhu Z, Liu B. Stromal fibroblasts in the microenvironment of gastric carcinomas promote tumor metastasis via upregulating TAGLN expression. BMC Cell Biol. 2013; 14:17.
10. Schellerer VS, Langheinrich M, Hohenberger W, Croner RS, Merkel S, Rau TT, Sturzl M, Naschberger E. Tumor-associated fibroblasts isolated from colorectal cancer tissues exhibit increased ICAM-1 expression and affinity for monocytes. Oncol Rep. 2014; 31:255–61.
11. Banerjee J, Mishra R, Li X, Jackson RS 2nd, Sharma A, Bhowmick NA. A reciprocal role of prostate cancer on stromal DNA damage. Oncogene. 2014; 33:4924–31.
12. Fausti F, Di Agostino S, Cioce M, Bielli P, Sette C, Pandolfi PP, Oren M, Sudol M, Strano S, Blandino G. ATM kinase enables the functional axis of YAP, PML and p53 to ameliorate loss of Werner protein-mediated oncogenic senescence. Cell Death Differ. 2013; 20:1498–509.
